# Room temperature charge-transfer phosphorescence from organic donor–acceptor Co-crystals†

**DOI:** 10.1039/d2sc03343g

**Published:** 2022-08-10

**Authors:** Swadhin Garain, Shagufi Naz Ansari, Anju Ajayan Kongasseri, Bidhan Chandra Garain, Swapan K. Pati, Subi J. George

**Affiliations:** New Chemistry Unit and School of Advanced Materials, Jawaharlal Nehru Centre for Advanced Scientific Research (JNCASR) Jakkur Bangalore 560064 India; Theoretical Sciences Unit, Jawaharlal Nehru Centre for Advanced Scientific Research (JNCASR) Jakkur Bangalore 560064 India

## Abstract

Engineering the electronic excited state manifolds of organic molecules can give rise to various functional outcomes, including ambient triplet harvesting, that has received prodigious attention in the recent past. Herein, we introduce a modular, non-covalent approach to bias the entire excited state landscape of an organic molecule using tunable ‘through-space charge-transfer’ interactions with appropriate donors. Although charge-transfer (CT) donor–acceptor complexes have been extensively explored as functional and supramolecular motifs in the realm of soft organic materials, they could not imprint their potentiality in the field of luminescent materials, and it still remains as a challenge. Thus, in the present study, we investigate the modulation of the excited state emission characteristics of a simple pyromellitic diimide derivative on complexation with appropriate donor molecules of varying electronic characteristics to demonstrate the selective harvesting of emission from its locally excited (LE) and CT singlet and triplet states. Remarkably, co-crystallization of the pyromellitic diimide with heavy-atom substituted and electron-rich aromatic donors leads to an unprecedented ambient CT phosphorescence with impressive efficiency and notable lifetime. Further, gradual minimizing of the electron-donating strength of the donors from 1,4-diiodo-2,3,5,6-tetramethylbenzene (or 1,2-diiodo-3,4,5,6-tetramethylbenzene) to 1,2-diiodo-4,5-dimethylbenzene and 1-bromo-4-iodobenzene modulates the source of ambient phosphorescence emission from the ^3^CT excited state to ^3^LE excited state. Through comprehensive spectroscopic, theoretical studies, and single-crystal analyses, we elucidate the unparalleled role of intermolecular donor–acceptor interactions to toggle between the emissive excited states and stabilize the triplet excitons. We envisage that the present study will be able to provide new and innovative dimensions to the existing molecular designs employed for triplet harvesting.

## Abstract 2

A modular, non-covalent donor–acceptor strategy is proposed to bias the excited-state manifold of organic systems and to realize unprecedented charge-transfer phosphorescence.

## Graphical abstract

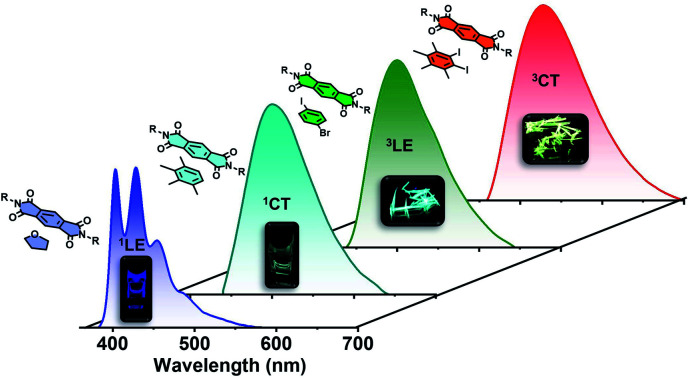

## Introduction

**Fig. 1:**
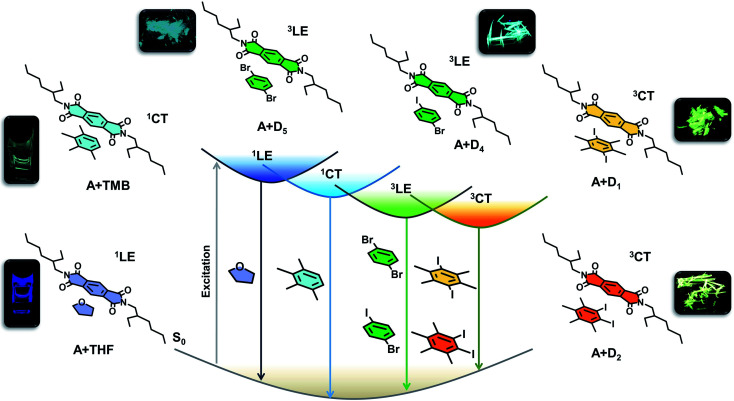
Schematic of the modular donor–acceptor co-assembly strategy to tune the excited state manifold of pyromellitic diimide phosphor. Molecular structure of PmDI (acceptor) and its tunable emission with different aromatic donors; locally excited fluorescence (^1^LE) in THF solution, charge-transfer (^1^CT) fluorescence in electron rich-aromatic solvents (*p*-xylene, mesitylene and TMB), locally excited phosphorescence (^3^LE) in 1-bromo-4-iodobenzene (D_4_), 1, 4-dibromobenzene (D_5_) and charge-transfer phosphorescence (^3^CT) in 1,4-diiodo-2,3,5,6-tetramethylbenzene (D_1_), 1,2-diiodo-3,4,5,6-tetramethylbenzene (D_2_). Simplified Jablonski diagram showing various emission processes possible in the excited state (photographs of ^1^LE, ^1^CT emission obtained by 340 nm and 370 nm Xe lamp excitation and ^3^LE and ^3^CT phosphorescent co-crystals under 365 nm UV lamp).

Biasing the landscape of electronic excited state manifolds of organic molecular systems has paramount importance in controlling various photophysical processes and resultant functions. The necessity to harvest triplet excitons owing to their practical implications in organic lighting devices, sensing, and bioimaging has triggered interest in manipulating the singlet and triplet excited states by innovative molecular designs.^1^ Thus, the recent past has witnessed a renaissance in the field of ambient triplet harvesting by effectively tuning the excited state dynamics with pertinent introduction of elegant molecular designs. Generally, the triplet excitons are harvested *via* two major photophysical processes; phosphorescence^2–4^ and thermally activated delayed fluorescence (TADF).^5^ Often, charge-transfer (CT) states play an important role in these ambient triplet harvesting processes, where these states can assist the exciton transfer by acting as an intermediate state or excitons can be directly harvested from the CT states.^5^ However, the molecular architecture with intramolecular charge transfer (ICT) characteristics is employed with fine modulation of optical properties for multiple applications. This includes enhanced circularly polarized luminescence of organic materials with high luminescence dissymmetry factor (|*g*_lum_|) endowed by increased electric transition dipole moment and amplified spontaneous emission of organic lasers by suppressing the ICT process.^6^ In the present contribution, we propose a modular approach to realize efficient triplet harvesting, by toggling between the locally excited (LE) and CT states of an organic molecule by through-space CT interactions, in non-covalent co-crystal scaffolds of donor and acceptor molecules (Fig. 1).

Supramolecular networks of CT co-crystals, with the co-facial organization of donor and acceptor chromophores,^7^ are an important class of organic functional materials and have been extensively explored in organic ferroelectrics^8^ and charge-transport.^9^ Further, the reversible dynamic nature of the CT interactions with tunable association constant, has been used for the design of various supramolecular materials, such as molecular motors,^10^ supramolecular polymers^11^ and foldamers.^12^ Despite the functional and supramolecular diversities offered by CT complexes, their optical properties are relatively less explored as they often form non-fluorescent CT complexes or charge-separated states.^13^ Even though emissive CT states (^1^CT) are reported, phosphorescence from triplet CT (^3^CT) states is rarely encountered.^14^ Although, attempts have been made to achieve phosphorescence from ^3^CT states under cryogenic conditions from CT complexes,^14^ to the best of our knowledge, there are no reports on phosphorescence from ^3^CT states based on organic ambient phosphors known to date.

Herein we introduce a modular donor–acceptor co-crystal approach to realize the unprecedented ambient CT phosphorescence. In this co-crystal approach, we envision that the excited state manifold can be modulated by the structural engineering of either donor and acceptor molecular components, and thereby selectively harnessing emission from LE or CT triplet excited states. This can be realized by the simultaneous incorporation of heavy atoms into the individual components, and/or tuning the acceptor or donor strength. The former results in populating the triplet state by enhancing spin–orbit coupling (SOC), and thereby inter-system crossing (ISC) efficiency by external or internal heavy atom effect,^1*a*^ while the latter prompts the excitons to preferentially channel into the CT states.^1*b*^ Further, we envisage that the co-crystal organization stabilized by various intermolecular interactions, would harvest the triplets by minimizing the common triplet quenching vibrational pathways.^1*a*,2*a*^

Earlier, we have unveiled that the anomalous exciplex like emission emanating from the arylene diimides in the presence of aromatic solvents have clear CT characteristics, resulting from the ground-state CT complexation between electron-rich aromatic solvents and electron-deficient arylene diimide cores.^15^ Thus, to toggle between different excited states by the CT strategy, we have chosen a donor–acceptor intermolecular architecture with arylene diimides based on pyromellitic diimide as acceptor and various electron rich heavy atom substituted benzene as donor components. Apart from the countless opportunities offered by arylene diimides to tune their electronic properties by structural modification,^16^ the added advantage bestowed by pyromellitic diimides due to their facile synthetic route and high triplet yields,^17^ urged us to continue exploring their potentials as the acceptor in the CT system. The simplified Jablonski diagram (Fig. 1) explains the possible electronic transitions realized by judiciously choosing the donor component to interact with pyromellitic diimide (PmDI). In presence of non-aromatic solvents like THF, PmDI exhibits its characteristic highly blue-shifted LE fluorescence (^1^LE). This can be further pushed into CT fluorescence with the help of electron rich aromatic solvents with low ionization potential that can form emissive CT complex with PmDI, as observed previously with naphthalene diimide derivatives.^15^ Remarkably, the integration of heavy atom into the donor counterpart induces ambient locally-excited (^3^LE) phosphorescence of PmDI*via* external heavy atom effect. More interestingly, the coalescence of both the above concepts, *i.e.*, introducing donor with strong electron-donating capacity with heavy atoms can trigger unique CT phosphorescence under ambient conditions, from the triplet ^3^CT states, as pictorially represented in the diagram (Fig. 1). Therefore, we illustrate tunable ambient phosphorescence from a simple arylene diimide derivative by selectively tuning the excited state dynamics of the phosphor using a modular non-covalent organic co-crystal approach.

## Results and discussions

**Fig. 2:**
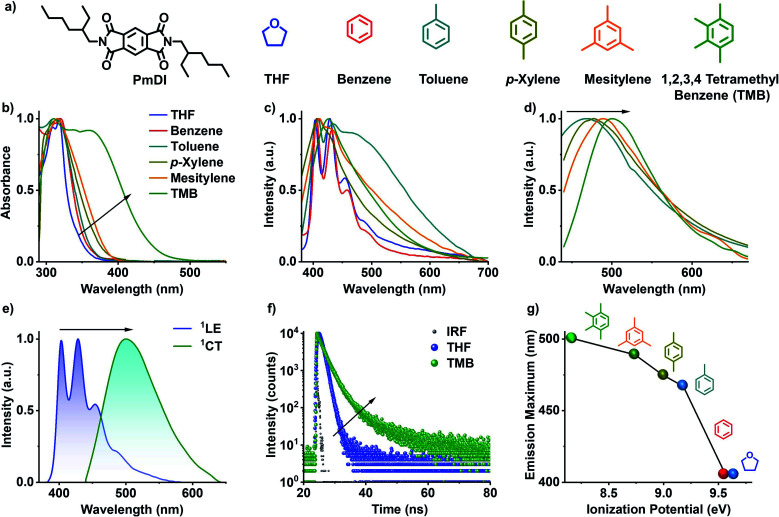
Solution state CT fluorescence of PmDI in electron rich aromatic solvents: (a) molecular structure of PmDI (acceptor) and different non-aromatic and aromatic solvents with varying electronic character. (b) Normalized absorption spectra of PmDI in various solvents, which shows the presence of ground state CT band in electron rich aromatic donors. (c) Normalized steady-state emission spectra showing ^1^LE emission in THF, benzene and broad, red-shifted CT emission band in electron-rich aromatic solvents (*λ*_exc_ = 340 nm). (d) Normalized emission spectra showing CT emission in electron-rich aromatic solvents upon selective excitation at the CT band (*λ*_exc_ = 420 nm). (e) Normalized emission spectra showing distinct ^1^LE emission in THF (*λ*_exc_ = 340 nm) and ^1^CT emission in TMB (*λ*_exc_ = 420 nm). (f) Fluorescence lifetime decay profiles of ^1^LE emission in THF (*λ*_exc_ = 340 nm, *λ*_collected_ = 420 nm) and ^1^CT emission in TMB (*λ*_exc_ = 442 nm, *λ*_collected_ = 500 nm), IRF is the instrument response function. (g) Plot of emission maximum *versus* the ionization potential of various aromatic solvents under study, which shows that emission maximum becomes red-shifted upon decreasing the ionization potential of the solvent.

Pyromellitic diimide (PmDI) derivative without any heavy-atom substitution was chosen as the acceptor component for the present study (Fig. 2a). Initially, the CT complexation of PmDI with various aromatic solvents was investigated by detailed spectroscopic studies. In THF, PmDI showed an absorption maximum of 320 nm and a structured emission with vibronic maxima at 420 nm, 440 nm and 460 nm, characteristic of local excited (^1^LE) fluorescence of PmDI monomer (Fig. 2b, c and e). Further investigation was carried out to unravel the differences in the spectroscopic properties of PmDI in the presence of various aromatic solvents which is crucial for the scope of the present investigation (Fig. 2c). As the electron donating capacity of the aromatic solvents was increased from benzene to 1,2,3,4-tetramethylbenzene (TMB), a new red-shifted absorption band appeared, with the maximum red-shift observed for TMB, which indicates the ground-state electronic interaction with the solvent molecules (Fig. 2b). The corresponding emission spectra upon exciting at the π–π* band (*λ*_exc_ = 320 nm) of PmDI in the same set of solvents exhibited dual emission bands, where the higher energy band corresponds to the ^1^LE state of PmDI, whereas the new broad red-shifted emission can be correlated to the ^1^CT emission (Fig. 2c and e). This is further confirmed by the emission spectra obtained upon the direct excitation of the lower energy band in the absorption spectra, which showed structureless emission with a gradual red-shifted maxima, with the increase in the donor strength of solvent, while moving from toluene to tetramethylbenzene (Fig. 2d). This is more evident from the plot of emission maximum *vs.* ionization potential where a nearly linear trend (from toluene to TMB) was observed confirming the CT nature of the interaction between PmDI and various aromatic solvents (Fig. 2g).^18^ Furthermore, the time-resolved lifetime decay experiments showed an average lifetime of 0.86 ns in THF (*λ*_exc_ = 340 nm, *λ*_collected_ = 420 nm) corresponding to the ^1^LE state of PmDI, and 2.56 ns in TMB (*λ*_exc_ = 442 nm, *λ*_collected_ = 500 nm), corresponding to the newly formed ^1^CT state (Fig. 2f and Table S1†). The increased lifetime in presence of TMB and other electron rich aromatic solvents, suggests the stabilization of the complex in electron donating solvents (Fig. 2f, S1 and Table S1†) which reiterated the CT nature of the red-shifted emission.

**Fig. 3:**
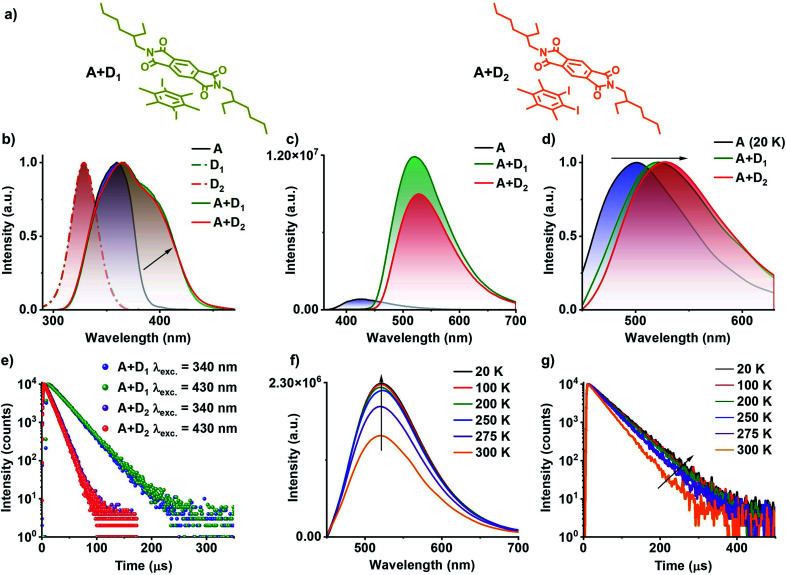
^3^CT phosphorescence studies of A+D_1_ and A+D_2_ co-crystals: (a) molecular structures for A+D_1_ and A+D_2_ donor–acceptor complexes. (b) Normalized excitation spectra of individual donors (D_1_ and D_2_, *λ*_monitored_ = 420 nm), acceptor (A, *λ*_monitored_ = 560 nm) and donor–acceptor co-crystal (A+D_1_ and A+D_2_, *λ*_monitored_ = 560 nm), which shows the red-shifted band for donor–acceptor co-crystal compared to individual components suggesting the formation of CT complex. (c) Steady-state emission spectra of the acceptor (A) and donor–acceptor co-crystal (A+D_1_ and A+D_2_), which shows the weakly emissive nature of bare acceptor and highly emissive nature of donor–acceptor pair (*λ*_exc_ = 340 nm). (d) Normalized delayed emission spectra of acceptor (A) doped in PMMA matrix at 20 K (1 wt% with respect to PMMA) and donor–acceptor co-crystal at room temperature, which shows a red-shift in the emission maximum of donor–acceptor pair compared to the ^3^LE emission of acceptor hinting towards the ^3^CT emission (*λ*_exc_ = 340 nm, delay time = 1 ms for A and 50 μs for A+D_1_ and A+D_2_). (e) Lifetime decay profile for A+D_1_ and A+D_2_ co-crystal upon excitation at 340 nm and selective excitation at CT band (*λ*_exc_ = 430 nm, *λ*_collected_ = 560 nm). Temperature-dependent (f) steady-state emission spectra and (g) lifetime decay profile (*λ*_collected_ = 560 nm) of A+D_1_ showing ^3^CT phosphorescence nature of the emission upon selective excitation at the CT band (*λ*_exc_ = 430 nm).

Inspired by the emissive singlet CT state formed between electron-rich aromatic solvents and PmDI, we made an attempt to realize phosphorescence from the ^3^CT state, by a donor–acceptor co-crystallization approach *via* modulating the donor characteristics, and thereby the intermolecular interactions. We have chosen the positional isomers based on electron-rich aromatic donor TMB with heavy atom substitution, *i.e.*, 1,4-diiodo-2,3,5,6-tetramethylbenzene (D_1_) and 1,2-diiodo-3,4,5,6-tetramethylbenzene (D_2_), to facilitate ISC and hence to stabilize the ^3^CT state (Fig. 3a).

Inspired by the emissive singlet CT state formed between electron-rich aromatic solvents and PmDI, we made an attempt to realize phosphorescence from the ^3^CT state, by a donor–acceptor co-crystallization approach *via* modulating the donor characteristics, and thereby the intermolecular interactions. We have chosen the positional isomers based on electron-rich aromatic donor TMB with heavy atom substitution, *i.e.*, 1,4-diiodo-2,3,5,6-tetramethylbenzene (D_1_) and 1,2-diiodo-3,4,5,6-tetramethylbenzene (D_2_), to facilitate ISC and hence to stabilize the ^3^CT state (Fig. 3a).

Preliminary studies of D_2_ with PmDI in solution state under cryogenic conditions exhibited a highly red-shifted band in the excitation spectra, suggesting the CT complexation (Fig. S2 and Table S2†). Therefore, 1 : 1 co-crystals of donor : acceptor (A+D_1_ and A+D_2_); where donor used is D_1_ or D_2_, and acceptor PmDI were grown, resulting in beautiful greenish-yellow crystals. Subsequent photophysical studies of the co-crystals exhibited an evident, red-shifted band in the excitation spectra monitored at 560 nm, incongruous to individual donor and acceptor spectral characteristics (Fig. 3b). The steady-state emission spectra when excited at 340 nm, showed an intense greenish-yellow emission with a maximum centred at 520 nm and 528 nm for A+D_1_ and A+D_2_, respectively, whereas respective individual donors (*Φ*_F_ of D_1_ and D_2_ is 0.72% and 0.65%, respectively) and acceptor (*Φ*_F_ = 1.1%) were weakly emissive (Fig. 3c). Notably, the gated emission spectra (delay time = 50 μs) of the co-crystals and emission lifetimes (*λ*_collected_ = 560 nm) of 22.02 μs and 12.24 μs for A+D_1_ and A+D_2_, respectively, collected under 340 nm excitation, pointed towards the delayed nature of the emission that emanates from them (Fig. 3e, S3 and Table S3†). Intensified emission and prolonged lifetime in vacuum compared to ambient conditions confirmed the role played by triplet state in the emission of these co-crystals (Fig. S4 and Table S3†). The nature of the delayed emission was further validated by temperature dependent studies (Fig. S5 and Table S4†). The increased emission intensity and lifetime upon decreasing the temperature confirms the phosphorescent nature of the emission (Fig. S5†). In order to get further insights into the nature of the emission, we further investigated photophysical characteristics of the individual components (Fig. S6†). Individual donors D_1_ and D_2_ in the crystal state were weakly emissive while the acceptor PmDI in PMMA matrix at 20 K and in THF at 77 K under cryogenic conditions showed a maximum around 500 nm, corresponding to the phosphorescence emission from LE triplet state of (^3^LE) of PmDI (Fig. S7, S8, Tables S5 and S6†). Remarkably, the delayed emission of the co-crystals is further red-shifted (A+D_1_ with *λ*_maximum_ at 520 nm and A+D_2_ with *λ*_maximum_ at 528 nm) and broad, which ruled out the possibility of the co-crystal emission to be originating from the acceptor ^3^LE emission and steered us to further investigate the role of CT states on the long-lived emission (Fig. 3d and S9†).

The key role played by CT process was investigated by selectively exciting at the CT band of the co-crystals at 430 nm (Fig. 3d and e). Similar emission spectral profiles and lifetime decay profiles observed upon selective excitation with that of the LE excitation at 340 nm, clearly substantiated the origin of long-lived emission to be the CT states formed by the donor–acceptor interactions (Fig. 3d and e). In addition, a closer examination of the excitation spectra showed a significant contribution from the CT band (shaded portion) which is much more red-shifted than the individual LE states of both D and A (Fig. 3b and S3†). Further, the temperature dependent studies carried out by the selective excitation at the CT band also displayed an increase in the emission intensity at lower temperature pointing towards phosphorescent nature of the CT emission (Fig. 3f, g, S10, Table S4†). Upon amalgamating both the observations, it can be inferred that the phosphorescence emission of these co-crystals are indeed originated from the ^3^CT state of the donor–acceptor complex, and to the best of our knowledge this is the first report on ^3^CT phosphorescence from organic co-crystal at room-temperature. The time-resolved emission and excitation experiments did not show any changes in the spectral maxima at various time intervals and wholly matched with the delayed emission spectra suggesting the exclusive contribution of the ^3^CT state in the phosphorescence emission (Fig. S11†). It is noteworthy to mention that the proposed CT co-crystal approach is very efficient to exclusively realize ^3^CT emission clearing out contributions from ^3^LE and ^1^LE states. Over and above to this, CT co-crystals exhibit exceptionally high phosphorescence quantum yields compared to the individual components and were measured to be 46% and 43% in air, and 71% and 65% under vacuum, for A+D_1_ and A+D_2_, respectively.

**Fig. 4:**
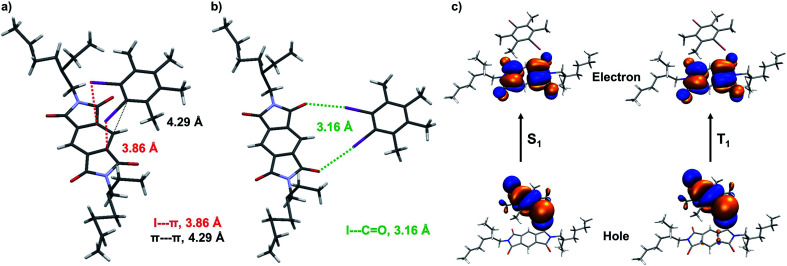
(a) Single-crystal X-ray diffraction data of A+D_2_ co-crystal: donor–acceptor arrangement of A+D_2_ pair, driven by various halogen bonding and weak π⋯π interactions, such as (a) I⋯π (marked with red lines), π⋯π (marked with black lines) in a stack and (b) I⋯C=O (marked with green lines) in the same plane. (c) Theoretical calculations of A+D_1_: natural transition orbitals (NTOs) of A+D_1_ pair for first excited singlet (S_1_) and triplet state (T_1_), calculated using TD-CAM-B3LYP level in conjunction with 6-31+g(d) basis set for C, N, O, H and LANL2DZ basis set showing CT character.

Further, we have attempted to characterize the molecular arrangement of donor and acceptor in the CT co-crystal by single-crystal X-ray diffraction (XRD) analysis (Fig. S12†). We have observed that the donor and acceptor are arranged in a slipped stacked manner where the donor is partially stacked on top of the acceptor (Fig. 4a and S13†). For example, in the case of A+D_2_, a weak π–π interaction (4.281 Å), is present between donor and acceptor (Fig. 4a and S13†). Interestingly, the two-iodine atoms of the donor (D_2_) face the π-surface of PmDI with a distance of 3.849 Å suggesting the presence of strong halogen–π interaction (Fig. 4a and S13†). Thus, the co-facial organization of the donor and acceptor components mediated by halogen–π and weak π–π interactions could be responsible for the intermolecular CT interaction observed in these co-crystals (Fig. 4a and S13†). The interdigitated nature of PmDI alkyl chains of adjacent D–A stacks suggests that hydrophobic interactions between alkyl chains also play a crucial role in the non-covalent organization of the co-crystals (Fig. S13†). Interestingly, we have observed another type of halogen bonding interaction between iodine and carbonyl with a distance of 3.161 Å within the same layer of donor–acceptor pair (Fig. 4b). We envisage that, these halogen bonding interactions in the co-crystal increase the SOC and ISC rate significantly and along with the CT interaction facilitate the efficient harvesting of triplet excitons from the ^3^CT state (Fig. 4a, b, S13, S14 and Table S7†).

The presence of through-space CT interaction between D_1_ (donor) and PmDI (acceptor) *via* their co-facial organization is further validated from the computed natural transition orbitals (NTOs) of the first excited singlet state (S_1_) where the hole is located on the D_1_ and electron on the π-surface of the acceptor (Fig. 4c, S14–S16†). The significant oscillator strength (*f* = 0.0023) with spatially separated highest occupied molecular orbitals (HOMO) and lowest unoccupied molecular orbitals (LUMO) leads to strong CT transition in the ground state (Fig. 4c, S14 and S15†). Interestingly, HOMO and LUMO of first excited triplet state (T_1_) were also located over donor and acceptor, respectively like the S_1_ state suggesting the CT nature of T_1_ state of A+D_1_ (Fig. 4c and S17†). The calculated spin–orbit coupling matrix element (SOCME) between S_0_ and T_1_ is 34.212 cm^−1^, which is significant enough to populate the triplet excitons to the ^3^CT state and thus helped to realize phosphorescence emission from a more thermodynamically stable state (Table S8†). Although the experimental energy gap between ^3^LE from ^3^CT is not very high, the presence of heavy atoms decreases the vibronic coupling, and thus the repopulation of ^3^LE from the ^3^CT state is not favourable. Further the significant energy difference (Δ*E*_ST_ = ∼110 meV) between ^1^CT (*λ*_maximum_ = 500 nm) and ^3^CT (*λ*_maximum_ = 528 nm) prevents the reverse intersystem crossing (RISC) to harvest triplets through the TADF pathway (Fig. 3d), thus leading to the exclusive ^3^CT pathway for the triplet harvesting.

We have further realized that the CT complexation can be indeed applied to a wide subset of electron-rich donors with heavy-atom incorporated into them to harvest the ^3^CT phosphorescence. For example, CT co-crystals of PmDI with another electron rich donor, with only two methyl groups and heavy atoms, 1,2-diiodo-4,5-dimethylbenzene (D_3_), also exhibited similar results although we could not solve the crystal structure (Fig. S17†). It is worth noticing that, due to the reduced electron-rich character of D_3_, compared to D_1_ or D_2_ the extent of CT strength was diminished, which is reflected as a blue-shift in the excitation spectrum and emission spectrum of A+D_3_ compared to that of the A+D_2_ cocrystal (Fig. S17†). Comparison of the respective maxima of ^3^LE phosphorescence of bare acceptor and ^3^CT phosphorescence A+D_3_ and A+D_2_ pair suggested that we can indeed modulate the ^3^CT phosphorescence emission by changing the donor strength (Fig. S17 and Table S9†), which would be worth exploring with a different series of electron rich donor molecules. The phosphorescence quantum yield of A+D_3_ is 42% in air.

**Fig. 5:**
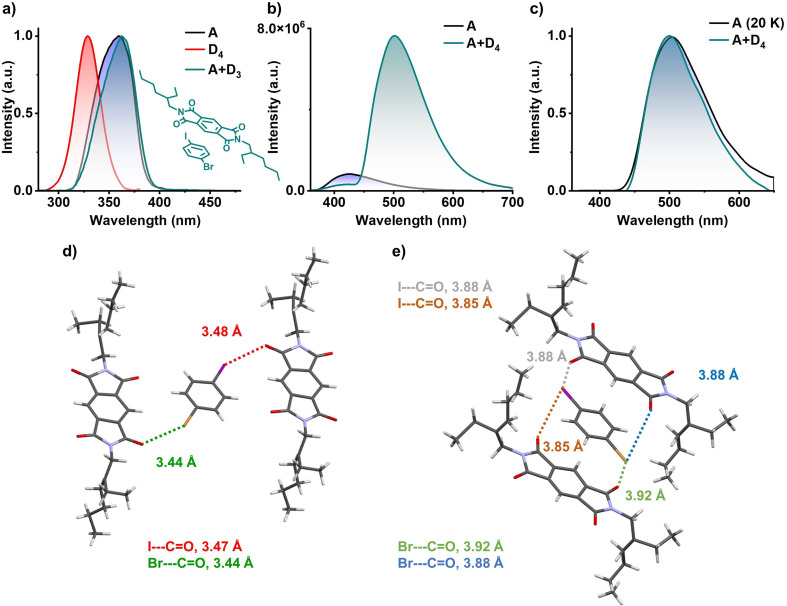
^3^LE phosphorescence studies and single-crystal X-ray diffraction data of A+D_4_ co-crystal: (a) excitation spectra of individual donor (D_4_, *λ*_monitored_ = 420 nm), acceptor (A, *λ*_monitored_ = 560 nm) and donor–acceptor co-crystal (A+D_4_, *λ*_monitored_ = 560 nm), which suggest absence of CT band (inset shows molecular structure for A+D_4_ pair). (b) Steady-state emission spectra of bare acceptor (A) and donor–acceptor pair (A+D_4_) shows weakly emissive nature of acceptor and highly emissive nature of donor–acceptor co-crystal (*λ*_exc_ = 340 nm). (c) Normalized delayed emission spectra of acceptor doped in PMMA matrix at 20 K (1 wt% with respect to PMMA) and donor–acceptor pair at room temperature show the same emission maximum, hinting towards the ^3^LE emission (*λ*_exc_ = 340 nm). Donor–acceptor arrangement of A+D_4_ pair, driven by halogen carbonyl interactions, (d) I⋯C=O (marked with red lines) and Br⋯C=O (marked with green lines) in the same plane, (e) I⋯C=O (marked with light-green and brown lines) and Br⋯C=O (marked with blue and orange lines) in the parallel plane.

Finally, in order to confirm the role of the electron rich aromatic donors to stabilize the ^3^CT state, we have utilized similar heavy atom substituted aromatic donor without any methyl substitution to decrease the donor strength (inset of Fig. 5a). Hence, we have grown 1 : 1 co-crystal of PmDI with 1-bromo-4-iodobenzene (D_4_) as the donor. In contrast to the previous observations, the excitation spectra of A+D_4_ co-crystal monitored at 560 nm did not show any red-shifted band and was similar to the excitation spectra of acceptor PmDI, pointing towards the absence of a CT complexation (Fig. 5a). The resulting bright green crystals exhibited intense emission upon exciting at 340 nm with a maximum at 500 nm, unlike weakly emissive individual donor and acceptor. The emission lifetime was measured out to be 0.38 ms (*λ*_exc_ = 340 nm, *λ*_collected_ = 560 nm), and the gated emission spectra (delay time = 0.1 ms) indicated the presence of delayed component in the emission which was proven out to be phosphorescence by the temperature dependent studies (Fig. 5b, c, S18 and Table S10†). Surprisingly, the gated emission spectra of the co-crystals entirely replicated the phosphorescence spectrum of PmDI in PMMA, collected at 20 K under 340 nm excitation, suggesting the source of origin of emission of A+D_4_ to be the LE triplet state (^3^LE) of the PmDI. Impressively, the phosphorescence quantum yield of A+D_4_ is 52% in air and 70% under vacuum, indicating a significant enhancement in the phosphorescence efficiency compared to that of PmDI alone which is weakly emissive in crystalline state.

Single crystal XRD analysis helped us to explain the reason behind the excellent ^3^LE phosphorescence efficiency of the A+D_4_ co-crystal (Fig. 5d, e, S19 and S20†). The co-crystals showed layers of self-sorted donor and acceptor molecules, which are alternatively spaced in the lateral direction, directed by the halogen–carbonyl interactions (iodo-carbonyl and bromo-carbonyl) between the molecules within a layer (Fig. 5d and S20†). In addition, four interlayer halogen–carbonyl interactions per donor molecules and, two each with the above and below layers, (together constituting a total of six halogen–carbonyl interactions) were also observed (Fig. 5e and S20†). We envisage that the presence of multiple halogen bonding interactions resulted in the significant enhancement of the SOC and ISC rate value *via* external heavy atom effect to achieve very high phosphorescence efficiency from ^3^LE state of PmDI (Fig. 5d, e and Table S7†). Further, in the absence of the significant CT interactions, A+D_4_ molecules adapt a two-dimensional lateral organization, rather than an alternatively stacked arrangement observed with A+D_2_.

Further, we have used 1,4-dibromobenzene derivative (D_5_) as a donor to deeply investigate the role of external heavy atom effect in triggering the ^3^LE phosphorescence. As expected, the emission spectra of A+D_5_ co-crystal was exactly similar to the ^3^LE phosphorescence of acceptor suggesting the origin of the phosphorescence emission to be ^3^LE in nature (Fig. S21 and Table S11†). Although we could not obtain the crystals for A+D_5_, powder XRD studies suggest that the packing of the donor and acceptor is similar in both A+D_5_ and A+D_4_ (Fig. S22†). Intriguingly, A+D_5_ showed a quantum yield of 16% in vacuum (11% in air), which is lesser than that of A+D_4_ (70% in vacuum and 52% in air), signifying the pivotal role of external heavy atom in inducing efficient phosphorescence emission (^3^LE). We infer that the presence of heavier iodine in A+D_4_ in the place of bromine increased the SOC, which resulted in efficacious emission with higher quantum yield. Furthermore, the theoretical calculations of A+D_5_ also supported the observed spectroscopic properties which showed that CT interactions are absent between the donor and acceptor molecules (Fig. S23–S25†). The computed NTOs showed that both the hole and electron are located on the π-surface of the acceptor for first excited singlet (S_1_) and triplet (T_1_) states, suggesting the LE transition (Fig. S24 and S25†).

## Conclusions

In summary, we have reported room-temperature ^3^CT phosphorescence from organic donor–acceptor co-crystals for the first time using pyromellitic diimide as the acceptor and heavy atom-substituted aromatic molecules as donors. The detailed spectroscopic studies and further analyses suggested that the donor–acceptor non-covalent complexation is an efficient modular approach to manipulate the electronically excited states of molecules. First, we have shown that PmDI (acceptor) can form ground-state CT complex with different aromatic solvents which has led to the CT fluorescence emission from the newly formed ^1^CT states in solution. Inspired from the potential of PmDI to form CT complexes and its effortless accessibility to triplet state, we further extrapolated the CT complexation of PmDI with electron-rich aromatic donors incorporated with heavy atoms to extract ^3^CT phosphorescence from the donor–acceptor complex under ambient conditions. The 1 : 1 co-crystal of PmDI with donors D_1_ and D_2_ exhibited an efficient greenish-yellow room-temperature phosphorescence emission from the CT states with appreciable quantum yields and lifetime augmented by the minimal vibrational dissipation of the triplet state and the enhanced rate of ISC facilitated by various intermolecular interactions between the donor and acceptor molecules in the long-range ordered alternate assembly. Although, very recently, a few examples on through-space CT based TADF have been reported,^19^ to the best of our knowledge, we have presented the first report on ^3^CT phosphorescence realized under ambient conditions. Later, we have switched the ^3^CT (*λ*_maximum_ = 528 nm) emission to ^3^LE (*λ*_maximum_ = 500 nm) emission, similar to neat PmDI by utilizing heavy-atom substituted donors D_4_ and D_5_, with reduced donor strength. In a concise, we can conclude that the supramolecular strategy based on through-space CT interactions, delineated in the current study can be cleverly used as tool to modulate between the various excited state manifolds of arylene diimide acceptors by the judicious choice of donors. We envisage that the current study also opens up an innovative way for generating ^1^CT and ^3^CT states by a supramolecular strategy for the molecular design of efficient TADF emitters unlike the conventional covalently linked systems. We believe that the present study has a momentous scope to harness triplet excitons from purely organic phosphors with remarkable efficiency, tunable emission and less synthetic efforts.

## Data availability

All the experimental and computational data were provided in the ESI.†

## Author contributions

SG carried out all the experiments directed by SJG analyzed the data and synthesized the molecules. SNA solved the crystal structure. BCG did the theory directed by SKP. SG, AAK and SJG wrote the manuscript with contributions from all authors.

## Conflicts of interest

There are no conflicts to declare.

## Supplementary Material

SC-013-D2SC03343G-s001

SC-013-D2SC03343G-s002
