# Supplementary material for: Room temperature charge-transfer phosphorescence from organic donor–acceptor Co-crystals
Source: Chem Sci. 2022 Aug 10;13(34):10011–9. doi: 10.1039/d2sc03343g (PMC9430718; doi:10.1039/d2sc03343g)
Supplement: SC-013-D2SC03343G-s001 [file SC-013-D2SC03343G-s001.pdf]

Electronic Supplementary Material (ESI) for Chemical Science.
This journal is © The Royal Society of Chemistry 2022

Supporting Information

Room Temperature Charge-Transfer Phosphorescence from Organic Donor-Acceptor Co-crystals

Swadhin Garain,[a] Shagufi Naz Ansari,[a] Anju Ajayan Kongasseri,[a] Bidhan Chandra Garain,[b] Swapan K. Pati,[b] and Subi J. George*[a]

[a.] New Chemistry Unit and School of Advanced Materials, Jawaharlal Nehru Centre for Advanced Scientific Research (JNCASR) Jakkur, Bangalore 560064, India

[b.] Theoretical Sciences Unit, Jawaharlal Nehru Centre for Advanced Scientific Research (JNCASR), Jakkur, Bangalore 560064, India.

Table of Contents

1. General Methods

2. Synthetic Schemes and Procedures

3. Experimental Procedures

4. Supporting Figures

5. Supporting Characterization Original Data

6. References

1. General Methods

NMR Measurements: ^{1}H and ^{13}C NMR spectra were recorded on a BRUKER AVANCE-400 fourier transformation spectrometer with 400 and 100 MHz respectively. The spectra were calibrated with respect to the residual solvent peaks. The chemical shifts are reported in parts per million (ppm) with respect to TMS. Short notations used are, s for singlet, d for doublet, t for triplet.

Spectroscopic Measurements: Electronic absorption spectra were recorded on Jasco V-750 UV-Visible spectrophotometer and emission spectra were recorded on FLS1000 spectrometer, Edinburgh Instruments. Solution state UV-Vis and emission spectra were recorded in 10 mm path length cuvette. Fluorescence spectra of the films were recorded in front-face geometry to avoid self-absorption.

Lifetime and quantum yield measurements: Fluorescence lifetimes were performed on a Horiba Delta Flex time-correlated single-photon-counting (TCSPC) instrument. A 340 nm diode and 373 nm, 442 nm laser diode with a pulse repetition rate of 1 MHz was used as the light source. The instrument response function (IRF) was collected by using a scatterer (Ludox AS40 colloidal silica, Sigma-Aldrich). Phosphorescence lifetime ($\lambda_{exc.}$ = 340 nm and 430 nm), gated emission and time-resolved excitation and emission were measured on FLS1000 spectrometer, Edinburgh Instruments equipped with a micro flash-lamp (µF2) set-up. Quantum yields were measured using an integrating sphere in the same instrument.

High Resolution Mass Spectrometry (HR-MS): HR-MS was carried out using Agilent Technologies 6538 UHD Accurate-Mass Q-TOFLC/MS.

Single Crystal X-ray Crystallography: Suitable single crystal of the **A+D$_2$** and **A+D$_4$** compound was mounted on a thin glass fibre with commercially available super glue. Intensity data were collected Bruker D8 VENTURE diffractometer equipped with a PHOTON detector and graphite-monochromated Mo-Kα radiation (λα = 0.71073 Å, 50 kV, 1mA) at 100 K. APEX III software was used to collect, reduce and integrate the raw data. The direct method was used for solving crystal structure, followed by full-matrix least-squares refinements against F2 (all data HKLF 4 format) using the SHELXL 2014/7 and difference Fourier synthesis and least-squares refinement revealed the positions of the non-hydrogen

atoms. All nonhydrogen atoms were refined anisotropically and remaining hydrogen atoms were placed in geometrically constrained positions and refined with isotropic temperature factors, generally 1.2 × Ueq of their parent atoms. Molecular structure drawings were prepared using the program Mercury (ver 3.1). We have selected the best crystal; however, few alerts are generated and can't be resolved despite several attempts due to the weak diffraction of crystals.

<u>Computational Details</u>: Ground State (S_0) of donor-acceptor pairs (**A+D$_5$**) was optimized using density functional theory (DFT). We are not able to optimize other donor-acceptor pairs. Excitation energies were calculated using B3LYP functional with 6-31+g(d) basis set except for Br and I.[S1,S2] For which we have used Lanl2dz functional with effective core potential. NTO calculations were done after TDDFT calculations. No solvent corrections were added in this work. The spin orbit coupling effect were considered to be a perturbation of scalar relativistic Kohn-Sham orbitals after SCF and TDDFT calculations (pSOC-TDDFT). The SOC matrix elements were calculated using B3LYP functional with a Slater type all-electron TZP basis set for all atoms as implemented in the ADF package.[S2-S6] While the excited state calculations were performed using B3LYP exchange-correlation functional with the same basis sets as mentioned before.

2. Synthetic Scheme and Procedure:

Pyromellitic dianhydride (PMDA) was purchased from Sigma Aldrich; Durene and 2-Ethylhexyl amine were purchased from Alfa-Aesar; 1-Bromo-4-iodobenzene and 1,4-Dibromobenzene were purchased from TCI, Acetic acid was purchased from Spectrochem and used without further purification.

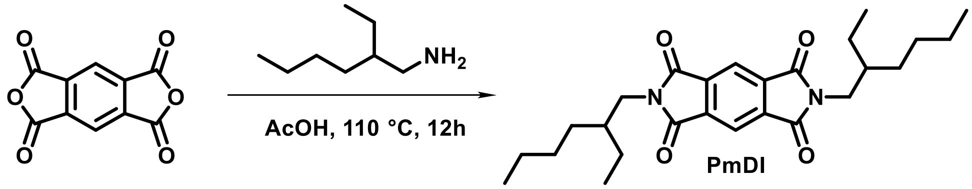

Scheme S1. Synthetic scheme for **PmDI** (Acceptor)

<u>PmDI:</u> Pyromellitic dianhydride (**1**) (0.50 g, 2.4mmol) was taken in a 100 mL round bottom flask and 25 mL acetic acid was added into it and stirred at room temperature for 30 minutes. 2-Ethylhexylamine (0.66 g, 5.1 mmol) was added to the reaction mixture and the reaction mixture was allowed to reflux for 12 hours. Water was added to the reaction mixture to get white precipitate. The precipitate was then filtered and dried under vacuum for 10 hours. Column chromatography was performed using chloroform as an eluent to get the pure product as white solid (0.86 g, 85 % yield). ^{1}H NMR ($CDCl_3$, 400 MHz), δ (ppm) = 8.26 (s, 2H), 3.64 (d, 4H, J = 7.2 Hz), 1.85 (t, 2H, J = 6 Hz), 1.55-1.26 (m, 16 H), 0.94-0.87 (m, 12

H); ^{13}C NMR ($CDCl_3$, 100 MHz), δ (ppm) = 166.6, 137.2, 118.2, 42.6, 38.3, 30.6, 28.5, 23.9, 23.00, 14.0 10.4 HRMS (APCI): *m/z* calculated for $C_{26}H_{36}N_2O_4$: 440.2675: observed 440.2760 $[M]^{\cdot+}$.

3. Experimental Procedures

We have used the following acceptors and donors for the ^{3}CT and ^{3}LE phosphorescence studies. The donors D_1[S7], D_2[S8] and D_3[S9] were synthesized according to the literature procedures.

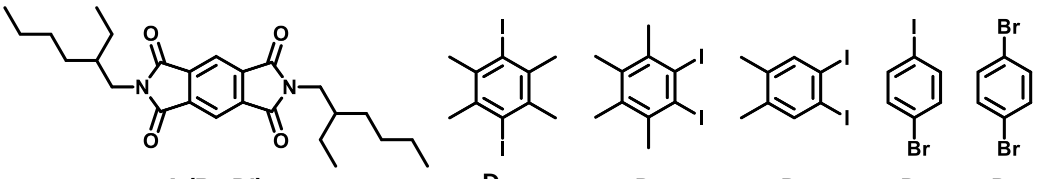

A (PmDI) **D_1** **D_2** **D_3** **D_4** **D_5**

Protocol for co-crystal synthesis: We have prepared saturated solutions of both donor and acceptors in chloroform and mixed them in a 1:1 molar ratio. Then acetonitrile was added into it as a bad solvent. The mixture was heated at 70 °C for ten minutes and then kept at room temperature for crystallization.

Protocol for sample preparation: All solution state studies were performed, keeping the final concentration of the samples to 0.05 mM and 0.1 mM from a stock solution of THF (1 mM). For thin films, acceptor molecules (1 mg) were mixed with 100 mg of PMMA. This mixture was then heated at 50 °C for 10 minutes followed by sonication (5 minutes) to dissolve all the components thoroughly. Then, 0.5 mL of this solution was drop-casted on a clean quartz substrate. Finally, the drop-casted thin films were dried at 60 °C for 30 minutes before performing the photophysical studies. For the phosphorescence studies of the co-crystal, a small amount of the co-crystal was placed in between two quartz plates.

4. Supporting Figures

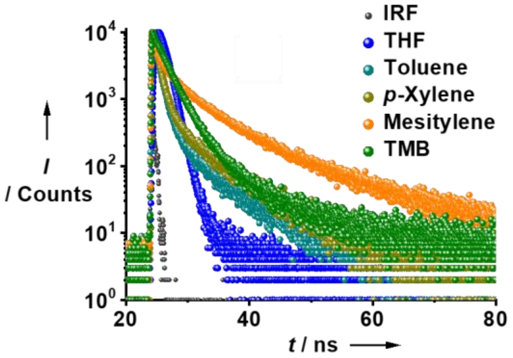

Figure S1. Fluorescence lifetime decay profiles of **PmDI** in various solvents: ^{1}LE emission in THF ($\lambda_{exc.}$ = 340 nm, $\lambda_{collected}$ = 420 nm), ^{1}CT emission in toluene, *p*-xylene, mesitylene ($\lambda_{exc.}$ = 404 nm, $\lambda_{collected}$ = 500 nm) and TMB ($\lambda_{exc.}$ = 442 nm, $\lambda_{collected}$ = 500 nm), IRF is the instrument response function ($\tau_{avg.}$ = 1.82 ns, 2.62 ns and 6.42 ns for toluene, *p*-xylene and mesitylene, respectively). Further details are mentioned in Table S1.

Note: Lifetime analyses show that ^{1}CT emission of **PmDI** in various aromatic solvents have a higher lifetime than the ^{1}LE emission in THF, further validating the CT nature of the broad emission band as a result of the CT complexation of **PmDI** with aromatic solvents.

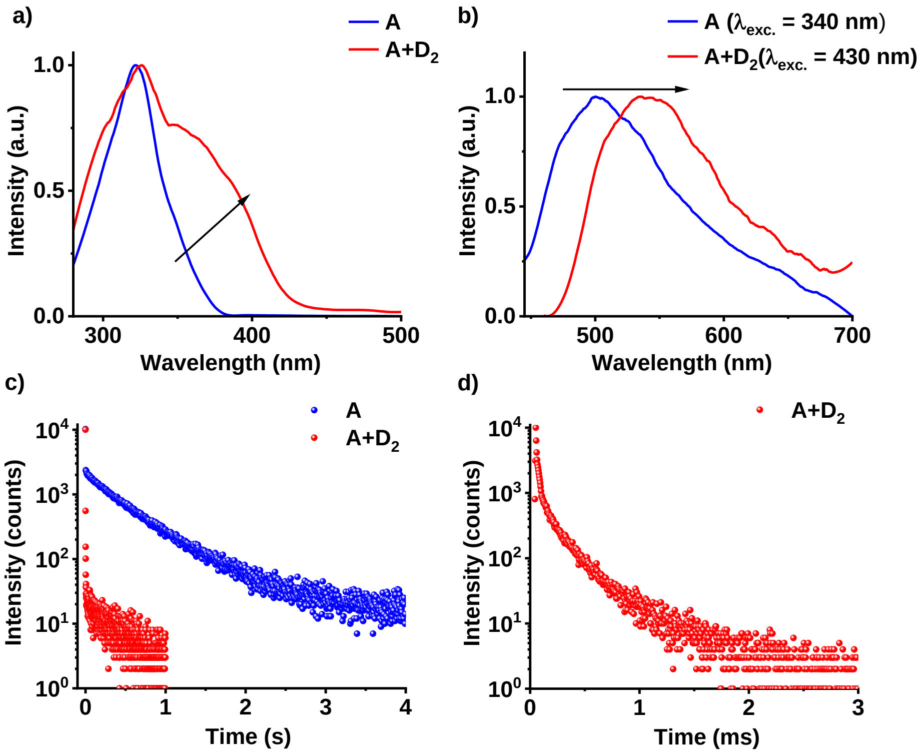

Figure S2. Comparison of the spectroscopic studies of **A+D₂** complex and **A** alone at 77 K in THF glassy matrix: a) Excitation spectra ($\lambda_{monitored}$ = 560 nm), b) delayed emission spectra (delay time = 0.5 ms). Corresponding lifetime decay profiles when excited at c) $\lambda_{exc.}$ = 340 nm, ($\tau_{avg.}$ = 0.48 s and for **A** alone and $\tau_{avg.}$ = 0.33 s (excluding fast component) for **A+D₂**), and d) $\lambda_{exc.}$ = 430 nm ($\tau_{avg.}$ = 0.16 ms). In both cases the emission collected at 560 nm. (Concentration of each component is 0.1 mM). Further details of lifetimes are mentioned in Table S2.

Note: a) Excitation spectra of **A+D₂** pair shows red-shifted band compared to the bare acceptor (**A)**, suggesting the formation of CT complex even in solution under cryogenic conditions. Emission spectrum of **A+D₂** gets red-shifted by 44 nm ($\lambda_{max.}$ = 544 nm) compared to **A** upon direct excitation at the CT band ($\lambda_{exc.}$ = 430 nm) pointing towards the CT nature of the phosphorescence emission. Unlike **A**, the sharp decay component in the lifetime decay plot of **A+D₂** implies the formation of another state different from that of ^{3}LE state, which is the ^{3}CT state. However, we have successfully extracted the newly formed state upon selectively exciting at the CT band, which clearly shows a short lifetime component compared to the ^{3}LE emission, and validated our hypothesis of ^{3}CT state formation.

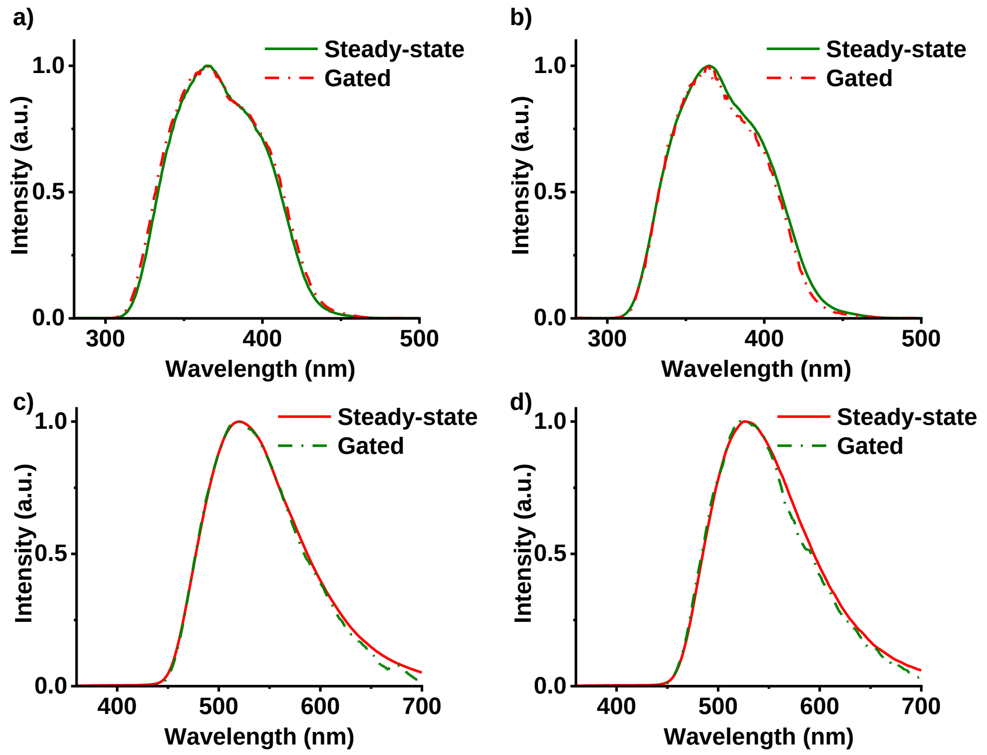

Figure S3. Normalized steady-state and delayed (delay time = 50 µs) excitation (above) and emission (below) spectra of a,c) **A+D$_1$** and b,d) **A+D$_2$** co-crystal ($\lambda_{exc.}$ = 340 nm $\lambda_{monitored}$ = 560 nm).

Note: The delayed excitation spectrum validates the long-lived nature of the CT process and its role in the origin of long-lived greenish-yellow emission of the co-crystals.

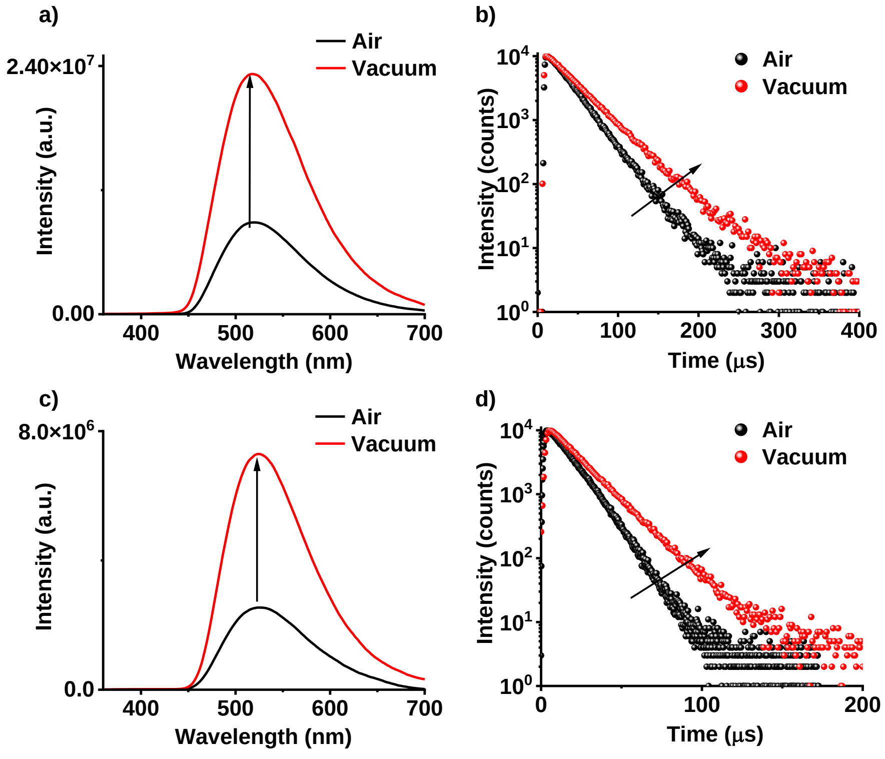

Figure S4. Spectroscopic studies of **A+D$_1$** and **A+D$_2$** co-crystals under air and vacuum: Steady-state emission spectra ($\lambda_{exc.}$ = 340 nm) of a) **A+D$_1$** and c) **A+D$_2$** and corresponding lifetime decay profiles ($\lambda_{exc.}$ = 340 nm, $\lambda_{collected}$ = 560 nm) of b) **A+D$_1$** (τ = 22.02 µs in air and 34.14 µs in vacuum) and d) **A+D$_2$** (τ = 12.24 µs in air and 17.39 µs in vacuum). Further details of lifetimes are mentioned in Table S3.

Note: Increase in the emission intensity and lifetime under vacuum confirms the presence of triplet contribution to the emission from the co-crystals.

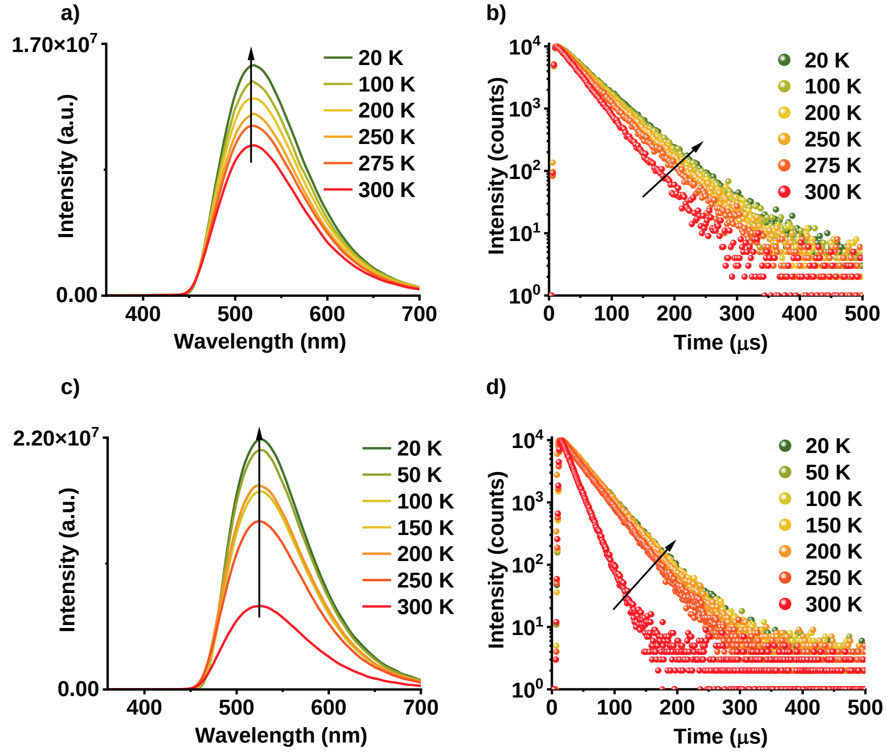

Figure S5. Temperature dependent studies of **A+D$_1$** and **A+D$_2$** co-crystals: Steady-state emission spectra ($\lambda_{exc.}$ = 340 nm) of a) **A+D$_1$** and c) **A+D$_2$**. Corresponding lifetime decay profiles ($\lambda_{exc.}$ = 340 nm, $\lambda_{collected}$ = 560 nm) of b) **A+D$_1$** (τ = 48.47 µs at 20 K and 24.12 µs at 300 K), and d) **A+D$_2$** (τ = 35.83 µs at 20 K and 17.39 µs at 300 K). Further details of lifetimes are mentioned in Table S4.

Note: The increase in the emission intensity (a,c) and lifetime (b,d) upon decreasing the temperature confirm the phosphorescence nature of the emission.

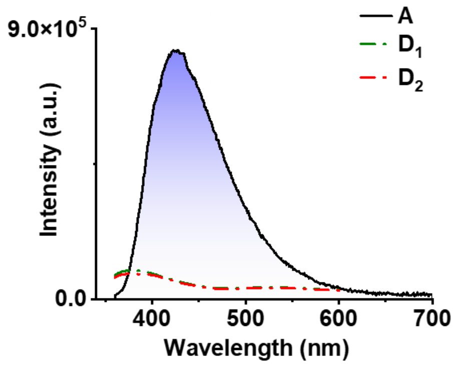

Figure S6. Steady-state emission spectra of individual donors (**D_1** and **D_2**), acceptor (**A**) shows weakly emissive nature of individual donors and acceptor ($\lambda_{exc.}$ = 340 nm).

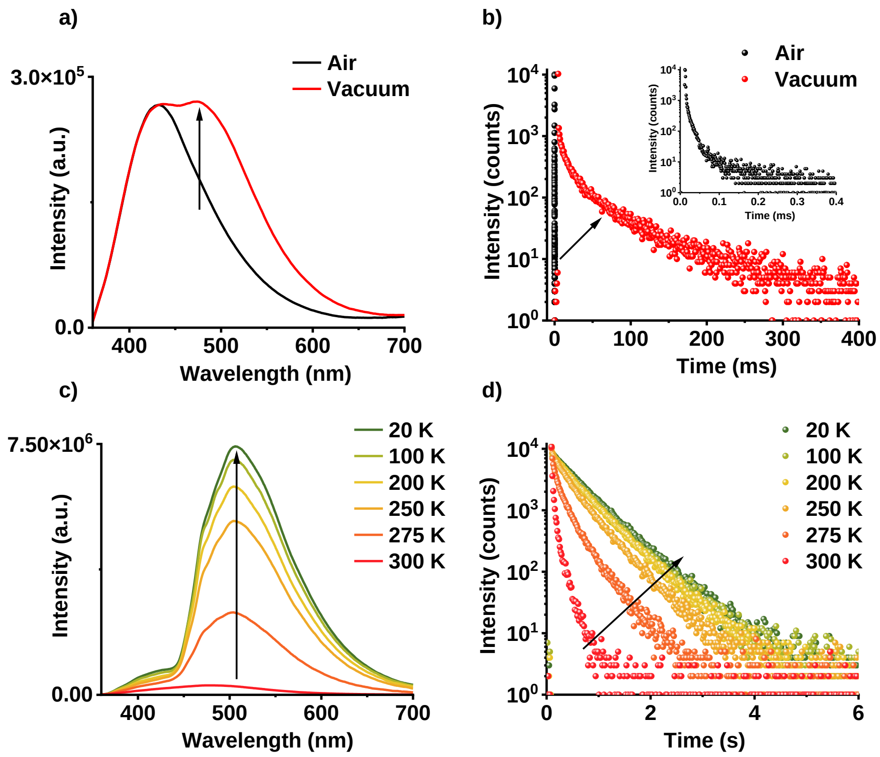

Figure S7. Spectroscopic studies under de-aerated conditions and temperature dependent studies of **A (PmDI)** in PMMA films (1 wt.% of **A** doped in PMMA matrix): a) Steady-state emission spectra ($\lambda_{exc.}$ = 340 nm) and b) lifetime decay profile ($\lambda_{exc.}$ = 340 nm, $\lambda_{collected}$ = 500 nm) under aerated and vacuum ($\tau_{avg.}$ = 7.72 µs in air and $\tau_{avg.}$ = 30.79 ms in vacuum). Inset represents the zoomed in lifetime decay profile in presence of air. c) Steady-state emission spectra ($\lambda_{exc.}$ = 340 nm) and d) lifetime decay profile ($\lambda_{exc.}$ = 340 nm, $\lambda_{collected}$ = 500 nm) in variable temperature measurements ($\tau_{avg.}$ = 525 ms at 20 K and 77 ms at 300 K). Further details of lifetimes are mentioned in Table S5.

Note: The increase in the (a) emission intensity and (b) lifetime under vacuum confirms the presence of triplet contribution to the emission. Further, an increase in the (c) steady-state emission intensity and

(d) lifetime upon decreasing the temperature confirms the locally excited (^{3}LE) phosphorescence nature of the **PmDI** emission in the absence of any donor molecules.

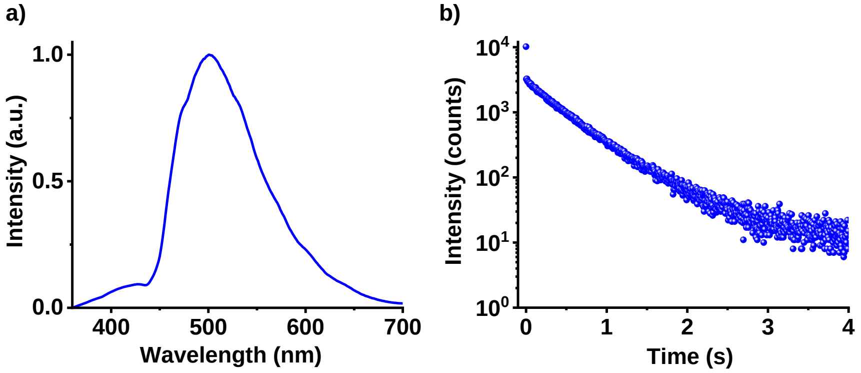

Figure S8. Phosphorescence study of **A** under cryogenic conditions (77 K in THF): a) Steady-state emission spectrum ($\lambda_{exc.}$ = 340 nm) and b) lifetime decay profile ($\lambda_{exc.}$ = 340 nm, $\lambda_{collected}$ = 500 nm, $\tau_{avg.}$ = 0.54 s) ([c] = 0.05 mM). Further details of lifetimes are mentioned in Table S6.

Note: Given steady-state emission spectrum and lifetime decay profile of the acceptor at very low concentration in THF glassy matrix is attested to the locally excited (^{3}LE) phosphorescence emission of **PmDI** monomer in the absence of any donor molecules.

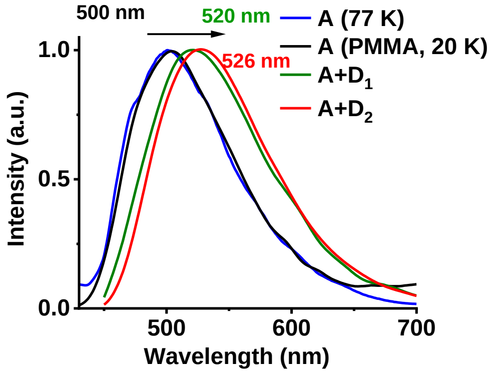

Figure S9. Comparison of ^{3}LE phosphorescence of **A** alone and ^{3}CT phosphorescence **A+D$_1$** and **A+D$_2$** co-crystals: Delayed emission spectra of **A** at cryogenic conditions (blue, [c] = 0.05 mM in THF), in PMMA matrix (black, 1 wt.% of **A** doped in PMMA matrix) shows ^{3}LE phosphorescence and **A+D$_1$** and **A+D$_2$** at ambient conditions show ^{3}CT phosphorescence (delay time = 1 ms for **A** and 50 ms for **A+D$_1$** and **A+D$_2$**, $\lambda_{exc.}$ = 340 nm for **A** and 430 nm **A+D$_1$** and **A+D$_2$**).

Note: Red-shifted and broad emission spectra of **A+D$_1$** and **A+D$_2$** co-crystals compared to the acceptor (**A**) phosphorescence emission suggests the ^{3}CT nature of the formers' emission.

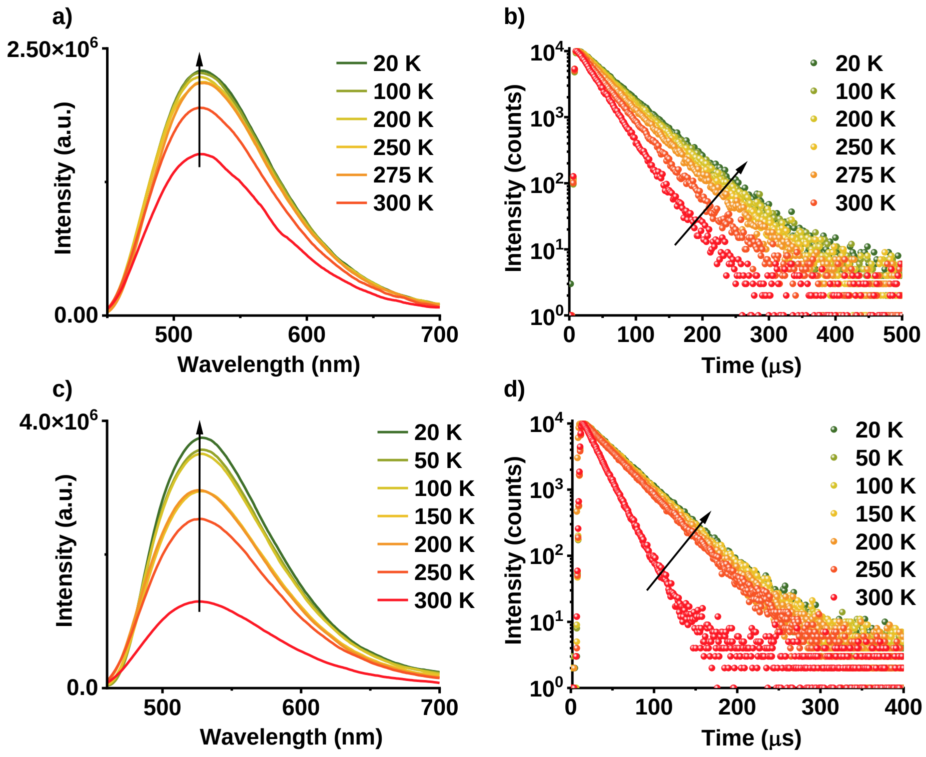

Figure S10. Temperature dependent studies of **A+D$_1$** and **A+D$_2$** co-crystals by the selective excitation at the CT absorption: Steady-state emission spectra ($\lambda_{exc.}$ = 430 nm) of a) **A+D$_1$** and c) **A+D$_2$**. Lifetime decay profiles ($\lambda_{exc.}$ = 430 nm, $\lambda_{collected}$ = 560 nm) of b) **A+D$_1$** (τ = 49.39 μs at 20 K and 35.67 μs at 300 K) and d) **A+D$_2$** (τ = 35.38 μs at 20 K and 17.33 μs at 300 K). Further details of lifetimes are mentioned in Table S4. **Note:** Increase in the (a) emission intensity and (b) lifetime upon decreasing the temperature on selective excitation at the CT band hints towards the phosphorescence nature of the CT emission.

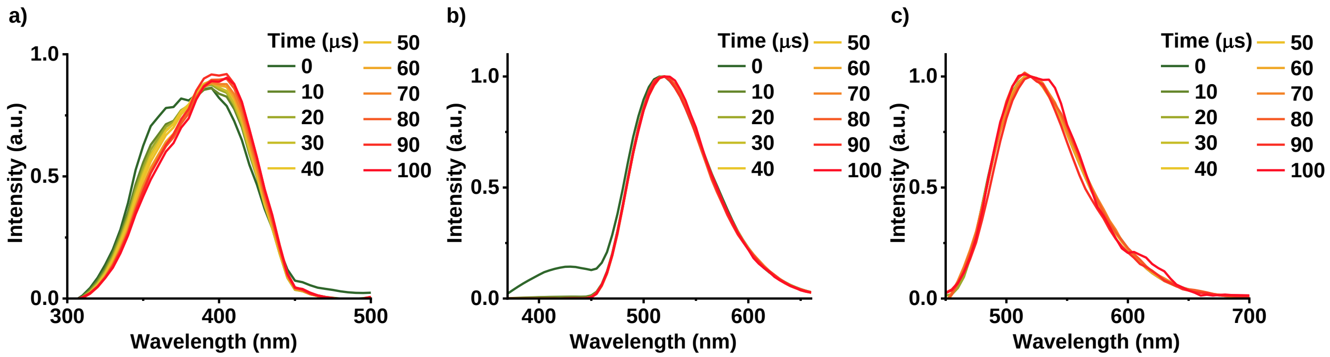

Figure S11. Time-resolved a) excitation spectra, ($\lambda_{monitored}$ = 560 nm), b) emission spectra upon exciting at $\lambda_{exc.}$ = 340 nm and c) upon selective excitation at the CT band ($\lambda_{exc.}$ = 430 nm) of **A+D$_1$** co-crystal.

Note: Time-resolved emission experiments indicate no spectral changes with gradual progress in time, suggesting that emission comes from the same excited state, i.e., ^{3}CT state and there is no transfer of excitons to other excited states.

	Calculated	Reported
Bond precision:	C-C = 0.0355 A	Wavelength=0.71073
Cell:	a=5.4537(5) alpha=90	b=38.527(4) beta=93.702(4) c=15.1042(15) gamma=90
Temperature:	300 K	
Volume	3167.0(5)	3167.0(5)
Space group	P 21/m	P 21/m
Hall group	-P 2yb	-P 2yb
Moiety formula	C26 H36 N2 O4, C24 H30 N2 O4, C10 H12 I2, 2(C H3)	?
Sum formula	C62 H84 I2 N4 O8	C3.35 H4.54 I0.11 N0.22 O0.16
Mr	1267.13	64.17
Dx,g cm-3	1.329	1.245
Z	2	37
Mu (mm-1)	1.046	1.036
F000	1308.0	1228.0
F000'	1306.59	
h,k,lmax	6,45,17	6,45,17
Nref	5692	5689
Tmin,Tmax	0.847,0.950	0.518,0.746
Tmin'	0.847	

Correction method= # Reported T Limits: Tmin=0.518 Tmax=0.746
AbsCorr = MULTI-SCAN

Data completeness= 0.999 Theta(max)= 24.999

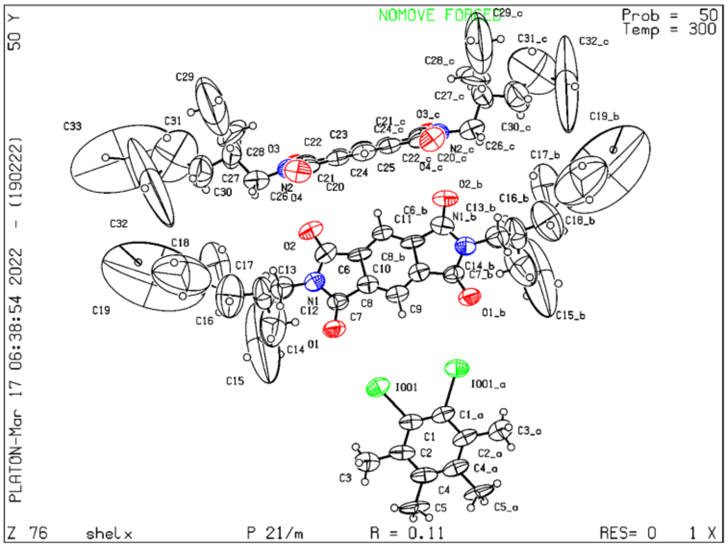

Figure S12. Structure data and cell parameters of **A+D$_2$** single crystal. CCDC Number is 2159661.

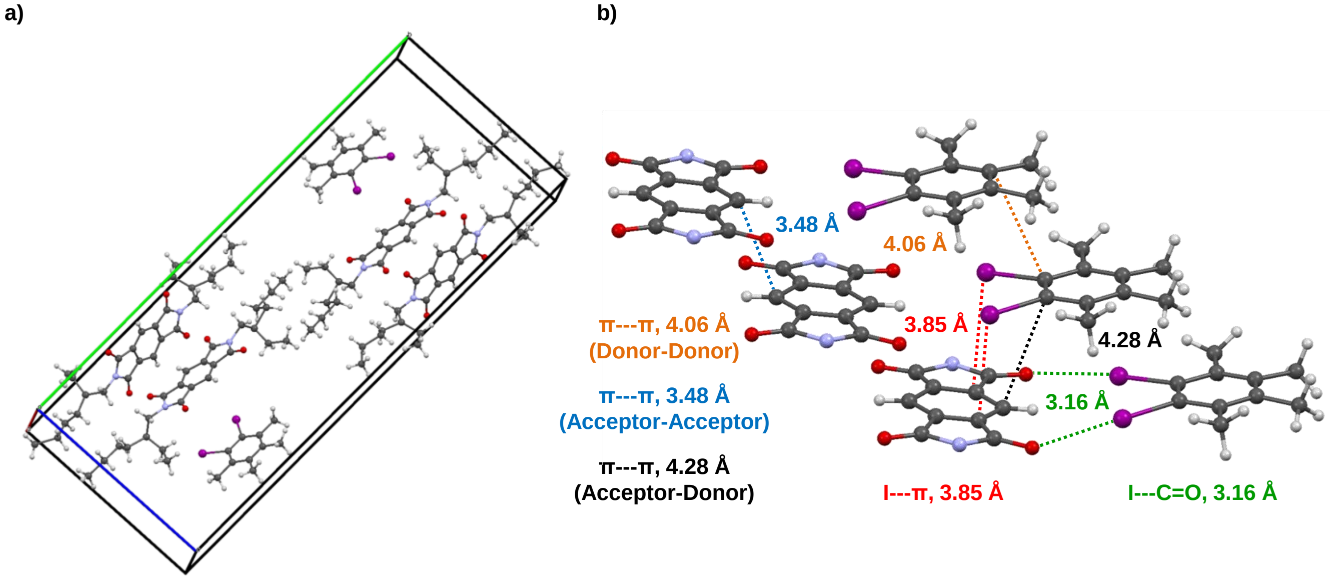

Figure S13. a**)** Unit cell and b) slipped stacked arrangement of acceptor and donor showing various intermolecular halogen-carbonyl interactions of **A+D$_2$** single crystal (we have removed ethyl hexyl chain for better illustration).

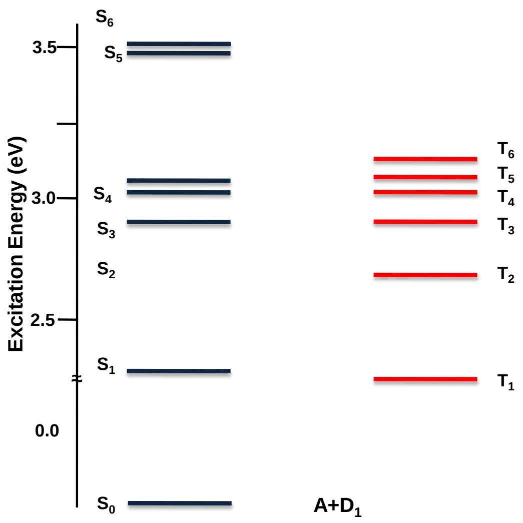

Figure S14. Excitation energies of **A+D$_1$** obtained from TDDFT calculation in the ground state geometry using B3LYP functional in conjunction with 6-31+g(d) basis set for C, H, N, O and LANL2DZ basis set for I with effective core potential.

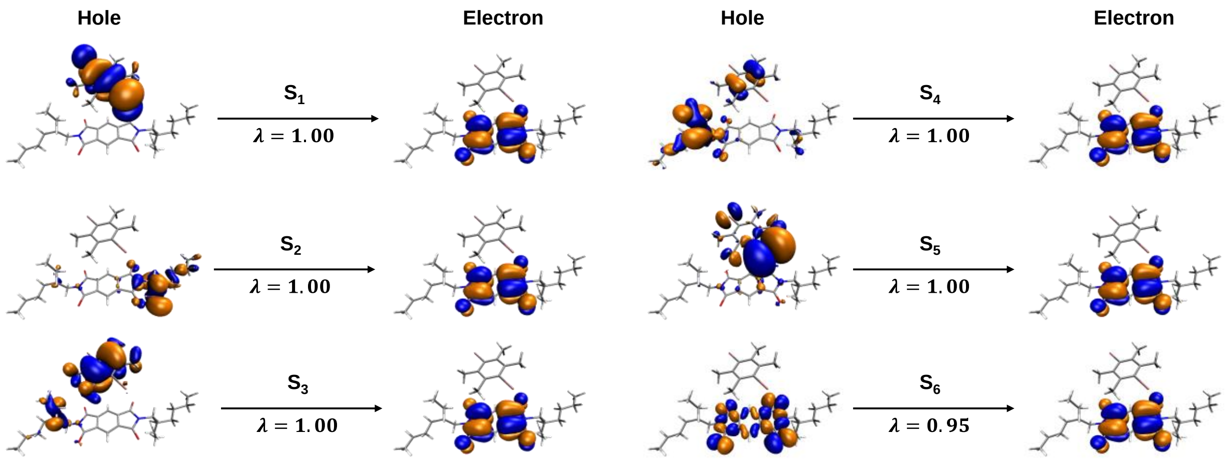

Figure S15. Natural Transition Orbitals (NTOs) for **A+D$_1$**. Singlet states are calculated at TD-B3LYP level with 6-31+g(d) basis set for C, H, O and N and LANL2DZ basis set for I with effective core potential, λ is the maximum value of singular value decomposition (SVD).

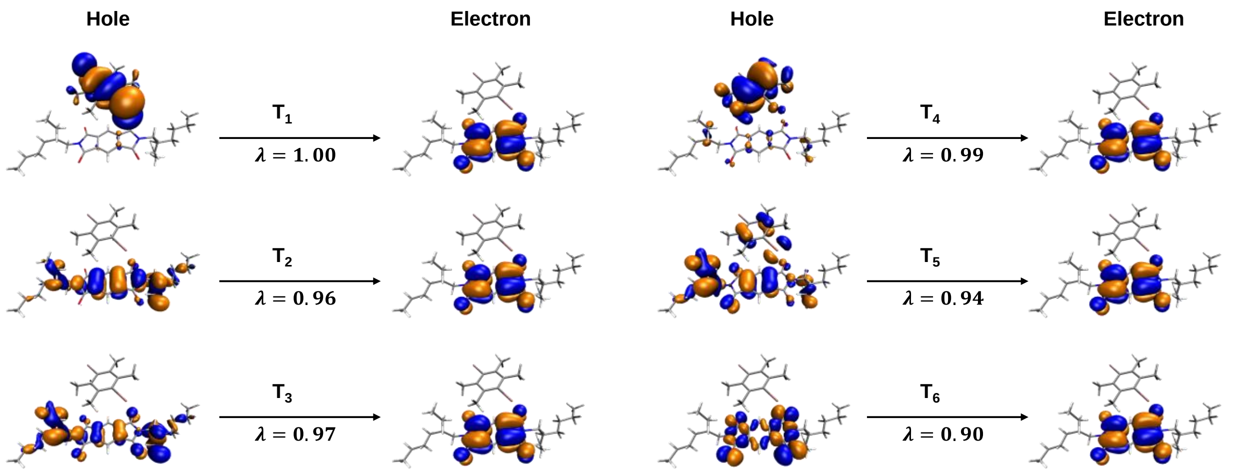

Figure S16. Natural Transition Orbitals (NTOs) for **A+D₁**. Triplet states are calculated at TD-CAMB3LYP level with 6-31+g(d) basis set for C, H, O and N and LANL2DZ basis set for Br and I with effective core potential, λ is the maximum value of singular value decomposition (SVD).

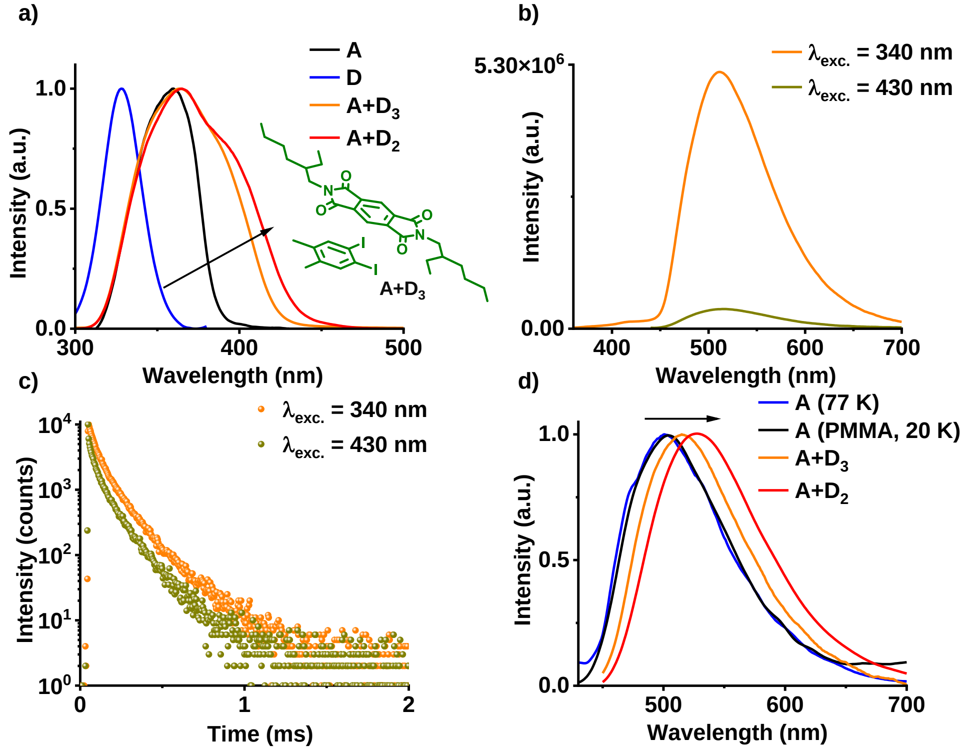

Figure S17. ^{3}CT phosphorescence studies of **A+D₃** co-crystals and comparison with the ^{3}LE phosphorescence of **A** alone and ^{3}CT phosphorescence **A+D₂** co-crystals: a) Excitation spectra of acceptor (**A**) alone and donor-acceptor co-crystal **A+D₂** (red line), **A+D₃** (orange line), red-shifted band of donor-acceptor co-crystal compared to the bare acceptor (**A**) phosphorescence emission suggests the formation of CT state ($\lambda_{monitored}$ = 560 nm). b) Steady-state emission spectra of donor-acceptor co-crystal (**A+D₃**). c) Corresponding lifetime decay profiles when excited at LE (λ_{exc} = 340 nm) and CT ($\lambda_{exc.}$ = 430 nm) band, in both cases the emission collected at 560 nm (τ_{avg} = 106.6 µs in air upon 340 nm excitation). d) Delayed

emission spectra of acceptor (**A**) alone at cryogenic conditions ([c] = 0.05 mM in THF) and in PMMA matrix (1 wt.% of **A** doped in PMMA matrix) show ^{3}LE phosphorescence, and **A+D$_2$** and **A+D$_3$** in air show ^{3}CT phosphorescence (delay time = 1 ms for **A** and 50 µs for **A+D$_2$** and **A+D$_3$**). Further details of lifetimes are mentioned in Table S7.

Note: Here, we have used a donor **D$_3$** with less electron-donating capability (two methyl groups are removed), which results in decrease in the extend of CT as evident from the excitation spectra. The CT band of **A+D$_3$** is blue-shifted compared to **A+D$_2$,** confirming reduced CT complexation in former when compared to latter. In addition, the comparison of the normalized delayed emission spectra (d) of **A** (^{3}LE), **A+D$_2$** and **A+D$_3$** (^{3}CT) reiterates the possibility of modulation of emission by varying the donor strength.

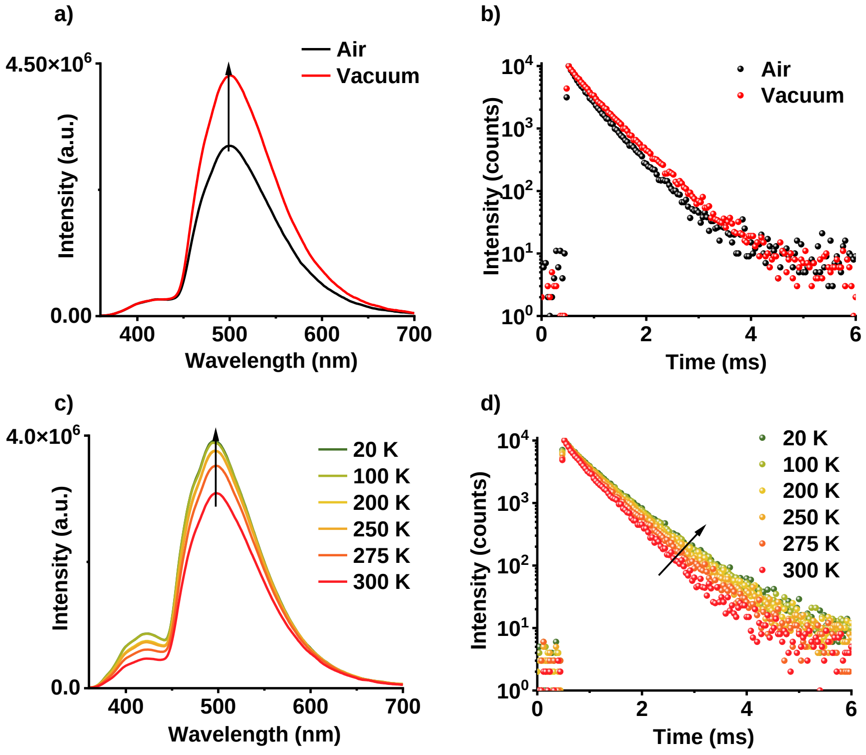

Figure S18. Spectroscopic studies in de-aerated conditions and temperature dependent studies of **A+D$_4$** co-crystal: a) Steady-state emission spectra ($\lambda_{exc.}$ = 340 nm) and b) corresponding lifetime decay profiles ($\lambda_{exc.}$ = 340 nm, $\lambda_{collected}$ = 560 nm) under air and vacuum (τ_{avg} = 0.38 ms in air and, 0.41 ms in vacuum). c) Steady-state emission spectra ($\lambda_{exc.}$ = 340 nm) and b) corresponding lifetime decay profile ($\lambda_{exc.}$ = 340 nm, $\lambda_{collected}$ = 560 nm, τ_{avg} = 0.61 ms at 20 K and 0.49 at 300 K) at different temperatures. Further details of lifetimes are mentioned in Table S8.

Note: Increase in the (a) emission intensity and (b) lifetime under vacuum confirms the presence of triplet contribution to the emission. Further, an increase in the (c) steady-state emission intensity and (d) lifetime upon decreasing the temperature confirms the phosphorescence nature of the emission.

Bond precision: C-C = 0.0225 A   Wavelength=0.71073

Cell: a=5.6900(4) b=8.3410(6) c=17.5111(12)
alpha=79.981(3) beta=82.483(2) gamma=89.991(2)

Temperature: 299 K

	Calculated	Reported
Volume	811.16(10)	811.16(10)
Space group	P 1	P 1
Hall group	P 1	P 1
Moiety formula	C26 H36 N2 O4, C6 H4 Br I	?
Sum formula	C32 H40 Br I N2 O4	C4 H5 Br0.12 I0.12 N0.25 O0.50
Mr	723.46	90.43
Dx,g cm-3	1.481	1.481
Z	1	8
Mu (mm-1)	2.255	2.255
F000	366.0	366.0
F000'	365.37	
h,k,lmax	6,9,20	6,9,20
Nref	5690[2845]	2845
Tmin,Tmax	0.805,0.854	0.518,0.746
Tmin'	0.595	

Correction method= # Reported T Limits: Tmin=0.518 Tmax=0.746
AbsCorr = MULTI-SCAN

Data completeness= 1.00/0.50   Theta(max)= 24.995

R(reflections)= 0.0471(2464)   wR2(reflections)= 0.1516(2845)

S = 1.083   Npar= 344

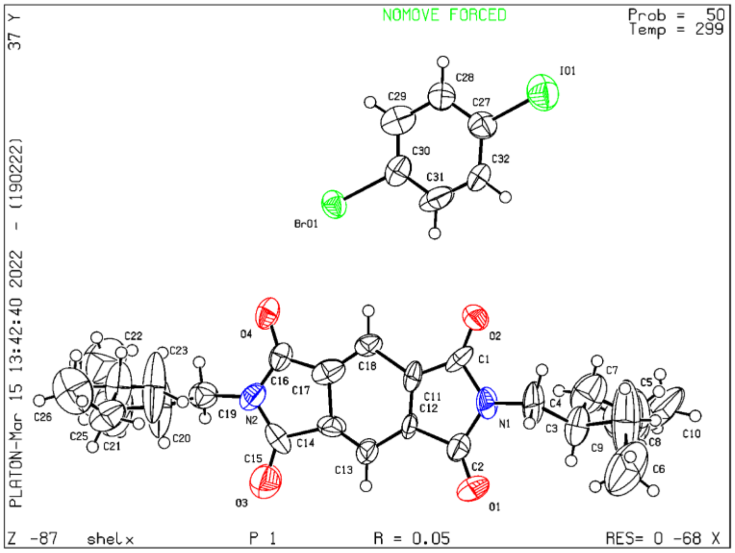

Figure S19. Structure data and cell parameters of **A+D$_4$** single crystal. CCDC Number is 2159217.

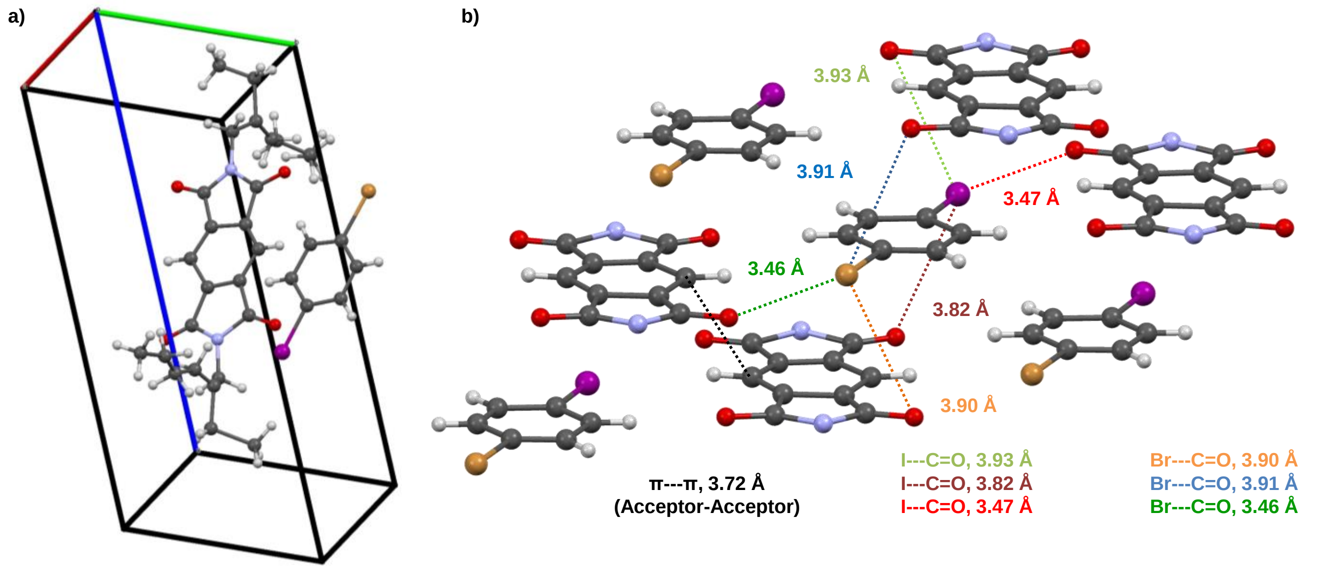

Figure S20. a**)** Unit cell and b) extended single crystal structure of **A+D$_4$** showing various intermolecular halogen bonding interactions (we have removed ethyl hexyl chain for better illustration).

Note: Six halogen bonding interactions (bromo-carbonyl and iodo-carbonyl) per donor molecule results in significant enhancement of the SOC value via external heavy atom effect to achieve very high phosphorescence efficiency from ^{3}LE state of **PmDI**.

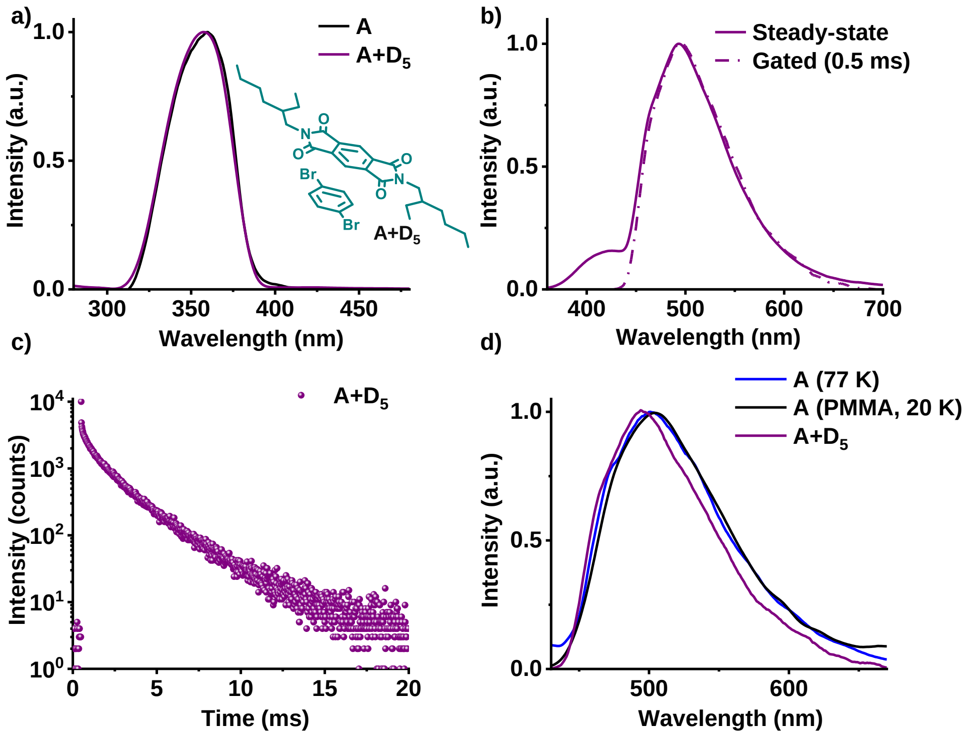

Figure S21. Phosphorescence studies of **A+D$_5$** co-crystal: a) Excitation spectra of the acceptor (**A**) alone and donor-acceptor co-crystal (**A+D$_5$**) ($\lambda_{monitored}$ = 560 nm), b) steady-state and gated emission spectra of **A+D$_5$** co-crystal ($\lambda_{exc.}$ = 340 nm, Inset: Photograph of **A+D$_5$** co-crystal under 365 nm UV lamp in air). c) Lifetime decay profile of **A+D$_5$** co-crystal ($\lambda_{exc.}$ = 340 nm, $\lambda_{collected}$ = 560 nm, τ_{avg} = 2.29 ms). d) Normalized delayed emission spectra of acceptor (**A**) alone at 77 K in THF, doped in PMMA matrix at 20 K (1 wt.% with respect to PMMA) and **A+D$_5$** at room temperature ($\lambda_{exc.}$ = 340 nm, delay time = 1 ms). Further details of lifetimes are mentioned in Table S9.

Note: Here, we have used 1,4-dibromobenzene as the donor (**D$_5$**), which shows lesser heavy-atom effect compared to donor **D$_4$**. The excitation spectrum of **A+D$_5$** is similar to **A** which indicates that no CT complexation is present in the former. Similarly, the gated emission spectra of **A** and **A+D$_5$** are exactly similar suggesting the origin of co-crystal emission to be the ^{3}LE phosphorescence of **PmDI**. However, the decreased heavy atom effect is reflected in the quantum yield measurement, where **A+D$_5$** shows a quantum yield of 11 %, much lesser than that of **A+D$_4$** (52 %), indicating the role of the heavy iodine atom in **A+D$_4$** co-crystal.

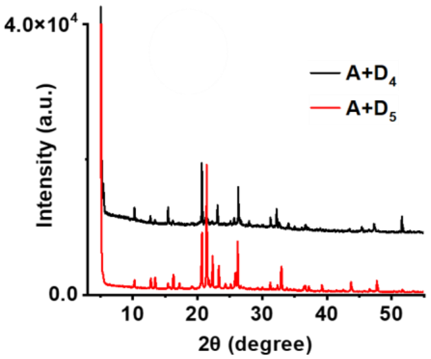

Figure S22. Powder XRD pattern of **A+D₄** and **A+D₅** co-crystals.

Note: PXRD suggests that the molecular packing of **A+D₄** and **A+D₅** co-crystals are similar.

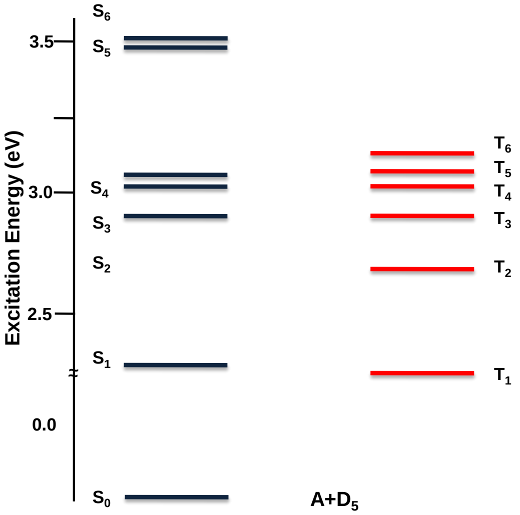

Figure S23. Excitation energies of **A+D₅** pair obtained from TDDFT calculation in the ground state geometry using B3LYP functional in conjunction with 6-31+g(d) basis set for C, H, N, O and LANL2DZ basis set for Br with effective core potential.

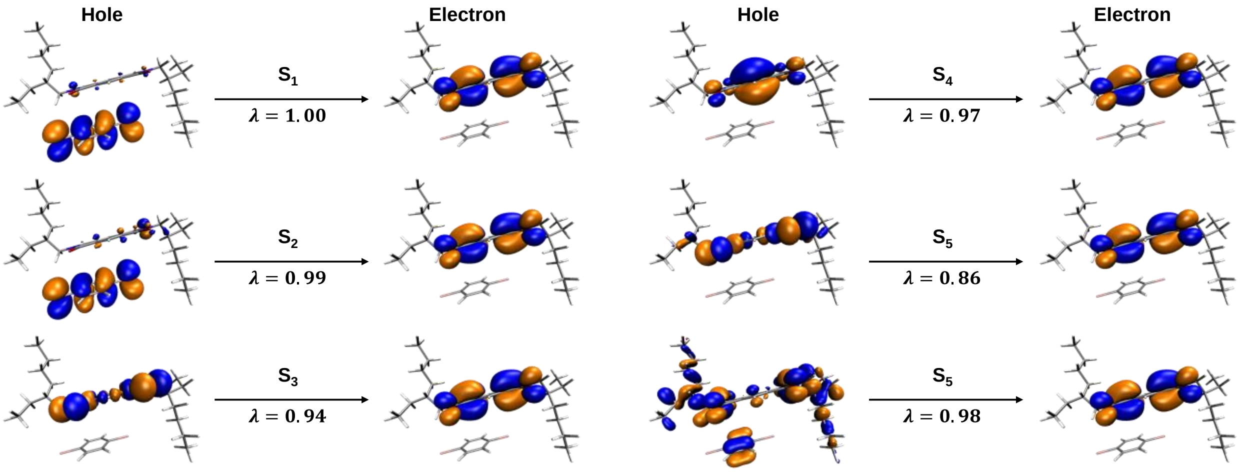

Figure S24. Natural Transition Orbitals (NTOs) for **A+D$_5$.** Singlet states calculated at TD-B3LYP level with 6-31+g(d) basis set for C, H, O and N and LANL2DZ basis set for I with effective core potential, λ is the maximum value of singular value decomposition (SVD).

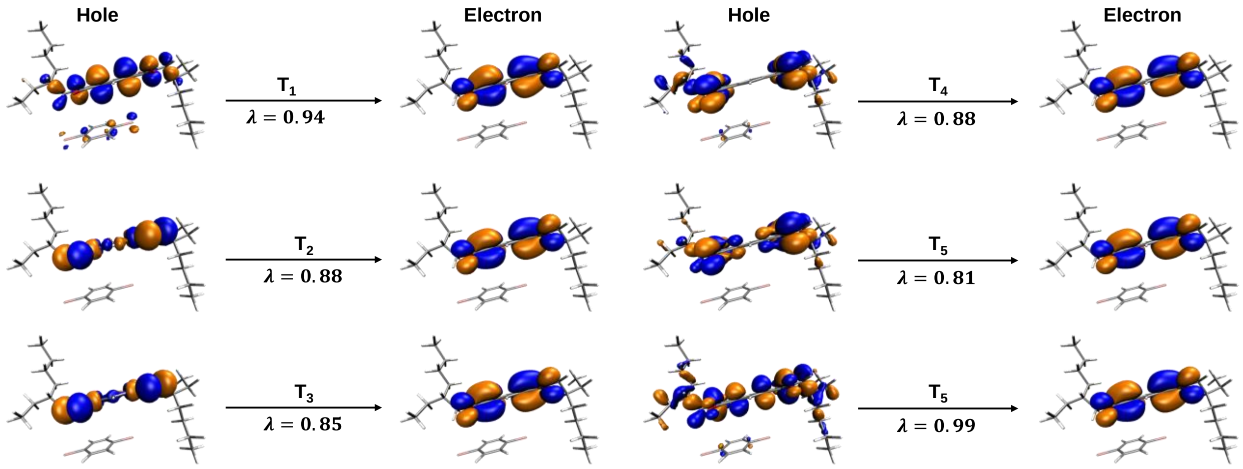

Figure S25. Natural Transition Orbitals (NTOs) for **A+D$_5$**. Triplet states calculated at TD-B3LYP level with 6-31+g(d) basis set for C, H, O and N and LANL2DZ basis set for Br and I with effective core potential, λ is the maximum value of singular value decomposition (SVD).

Note: The absence of CT complexation between **D$_5$** with the **PmDI** core (acceptor) is clear from the computed natural transition orbitals (NTOs) of the first excited singlet (S_1) and triplet (T_1) states, where the hole and electron both are located on the π-surface of the acceptor, suggesting towards the LE transition.

Table S1. Summary of fluorescence decay of **PmDI** (**A**) in various solvents.

Solvents	$\lambda_{exc.}$(nm)	$\lambda_{collected}$ (nm)	τ_1 (ns)	τ_2 (ns)	τ_3 (ns)	$<\tau_{avg}>$ (ns)
THF	340	420	0.80 (95%)	2.00 (5%)	-	0.86
Toluene	404	500	0.53 (38%)	1.25 (48%)	7.29 (14%)	1.82
p-Xylene	404	500	2.52 (20%)	0.74 (61%)	8.78 (19%)	2.62
Mesity-lene	404	500	3.10 (45%)	10.63 (47%)	0.41 (8%)	6.42
TMB	442	500	0.37 (12%)	2.16 (79%)	9.00 (9%)	2.56

Table S2. Summary of phosphorescence decay of **A+D$_2$** complex and **A** alone at 77 K in THF glassy matrix.

	$\lambda_{exc.}$(nm)	$\lambda_{collected}$ (nm)	τ_1 (s)	τ_2 (s)	τ_3 (s)	$<\tau_{avg.}>$ (s)
**A**	340	560	0.28 (27%)	0.56 (73%)	-	0.48
**A+D$_2$**	340	560	0.33 (100 %)	-	-	0.33
**A+D$_2$**	430	560	0.016 ms (32%)	0.11 ms (39 %)	0.37 ms (29%)	0.16 ms

Table S3. Summary of phosphorescence decay of **A+D$_1$** and **A+D$_2$** co-crystals under air and vacuum.

	$\lambda_{exc.}$(nm)	$\lambda_{collected}$ (nm)	τ (µs)
**A+D$_1$** in Air	340	560	22.02 (100%)
**A+D$_1$** in Vac-uum	340	560	34.14 (100%)
**A+D$_2$** in Air	340	560	12.24 (100 %)
**A+D$_2$** Vacuum	340	560	17.39 (100%)

Table S4. Summary of phosphorescence decay of **A+D$_1$** and **A+D$_2$** co-crystals in different temperature.

	$\lambda_{exc.}$(nm)	$\lambda_{collected}$ (nm)	τ (µs) at 20 K	τ (µs) at 300 K
**A+D$_1$**	340	560	48.47 (100%)	34.12 (100%)
**A+D$_1$**	430	560	49.39 (100%)	35.67 (100%)
**A+D$_2$**	340	560	35.83 (100 %)	17.39 (100%)
**A+D$_2$**	430	560	35.38 (100%)	17.33 (100%)

Table S5. Summary of phosphorescence decay of **PmDI,** doped in PMMA matrix (1 wt.% with respect to PMMA) in de-aerated conditions and in different temperature.

	$\lambda_{exc.}$(nm)	$\lambda_{collected}$ (nm)	τ_1 (ms)	τ_2 (ms)	τ_3 (ms)	$<\tau_{avg.}>$ (ms)
Air	340	500	1.09 µs (66 %)	10.35 µs (28 %)	68.37 µs (5 %)	7.72 µs
Vacuum	340	500	4.28 (10 %)	19.57 (43 %)	77.86 (47 %)	30.79
20 K	340	500	340 (22%)	590 (78 %)	-	535
300 K	340	500	30.80 (60 %)	147 (40 %)	-	77.28

Table S6. Summary of phosphorescence decay of **PmDI** (**A**) under cryogenic conditions (77 K in THF, [c] = 0.05 mM).

	$\lambda_{exc.}$(nm)	$\lambda_{collected}$ (nm)	τ_1 (s)	τ_2 (s)	τ_3 (s)	$<\tau_{avg.}>$ (s)
77 K	340	500	0.298 (26 %)	0.595 (78 %)	-	0.54

Table S7. Summary k_{P^r}(s^{-1}), k_{ISC}(s^{-1}), and k_{nr}(s^{-1}) of **A+D₁**, **A+D₂**, **A+D₃**, **A+D₄** and **A+D₅** co-crystals.

Co-crystals	Φ_P	$<\tau>_F$	$k_{ISC} = \Phi_P/<\tau>_F$
**A+D$_1$**	46 %	13.3 ns ($\lambda_{exc.}$ = 442 nm, $\lambda_{collected}$ = 460 nm)	3.46 x 10^{-9} s^{-1}
**A+D$_2$**	43 %	13.3 ns ($\lambda_{exc.}$ = 442 nm, $\lambda_{collected}$ = 460 nm)	3.23 x 10^{-9} s^{-1}
**A+D$_3$**	42 %	13.4 ns ($\lambda_{exc.}$ = 442 nm, $\lambda_{collected}$ = 460 nm)	3.13 x 10^{-9} s^{-1}
**A+D$_4$**	52 %	1.8 ns ($\lambda_{exc.}$ = 373 nm, $\lambda_{collected}$ = 430 nm)	2.88 x 10^{-8} s^{-1}
**A+D$_5$**	11 %	1.3 ns ($\lambda_{exc.}$ = 442 nm, $\lambda_{collected}$ = 430 nm)	8.46 x 10^{-9} s^{-1}

Table S8. Summary of phosphorescence decay of **A+D$_3$** co-crystal.

Co-crystals	$<S\|H_{SO}\|T>$	T_1
**A+D$_1$**	S_0	34.21
**A+D$_2$**	S_0	63.94
**A+D$_4$**	S_0	17.73
**A+D$_5$**	S_0	10.45

Table S9. Summary of phosphorescence decay of **A+D$_3$** co-crystal.

$\lambda_{exc.}$(nm)	$\lambda_{collected}$ (nm)	τ_1 (µs)	τ_2 (µs)	<$\tau_{avg.}$> (µs)
340	560	47.60 (47 %)	159 (53 %)	106.64
430	560	33.10 (39 %)	132 (61 %)	93.43

Table S10. Summary of phosphorescence decay of **A+D$_4$ co-crystals** in de-aerated conditions and in different temperature.

	$\lambda_{exc.}$(nm)	$\lambda_{collected}$ (nm)	τ_1 (ms)	τ_2 (ms)	τ_3 (ms)	<$\tau_{avg.}$> (ms)
Air	340	500	0.22 (35 %)	0.47 (65 %)	-	0.38
Vacuum	340	500	0.19 (28 %)	0.49 (72 %)	-	0.406
20 K	340	500	0.026 (14 %)	0.668 (85 %)	3.32 (1 %)	0.61
300 K	340	500	0.17 (10 %)	0.51 (89 %)	1.64 (1 %)	0.49

Table S11. Summary of phosphorescence decay of **A+D$_5$** co-crystals.

$\lambda_{exc.}$(nm)	$\lambda_{collected}$ (nm)	τ_1 (ms)	τ_2 (ms)	τ_3 (ms)	<$\tau_{avg.}$> (ms)
340	560	0.31 (8 %)	1.36 (52 %)	3.0 (52 %)	2.29

5. Supporting Characterization Original Data

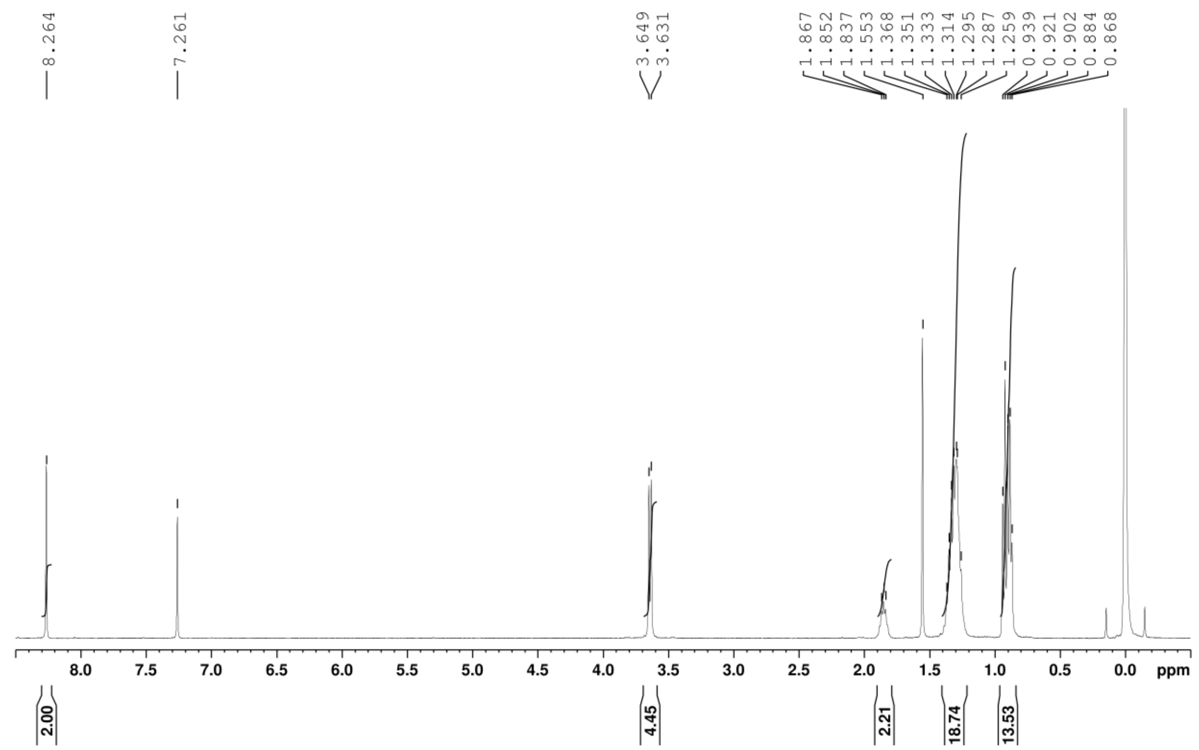

Figure S26. ^{1}H NMR spectrum of **PmDI** in $CDCl_3$.

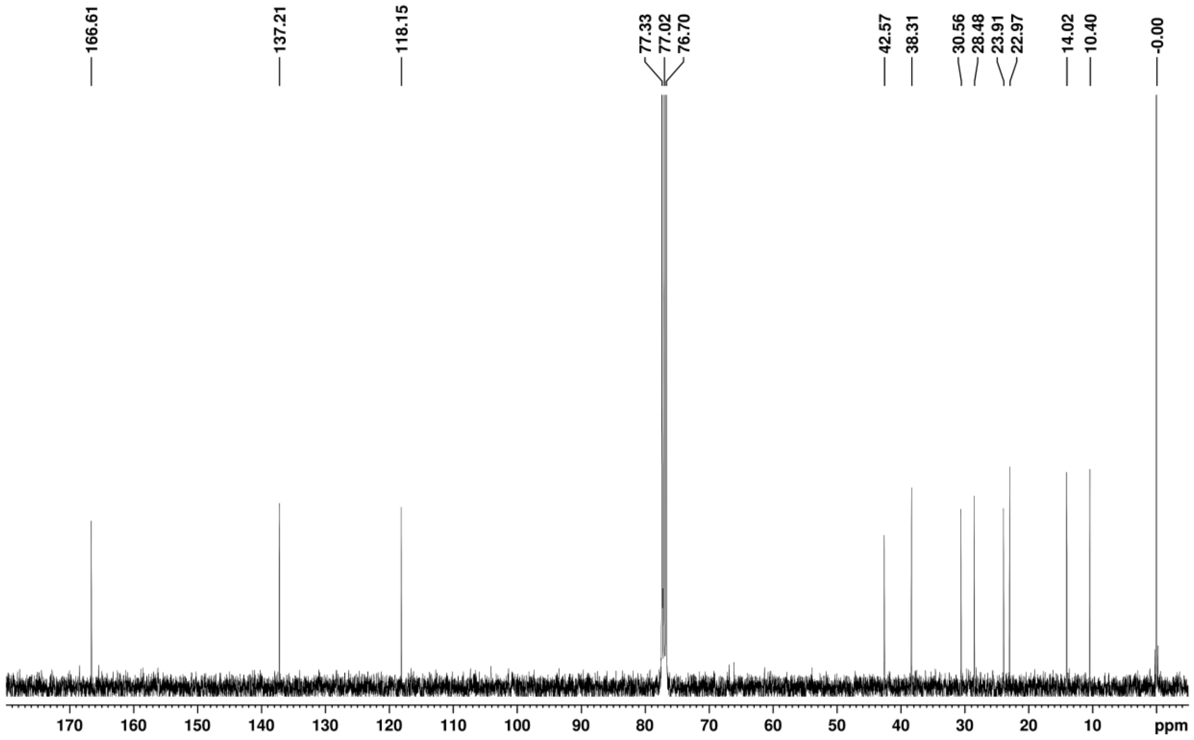

Figure S27. ^{13}C NMR spectrum of **PmDI** in $CDCl_3$.

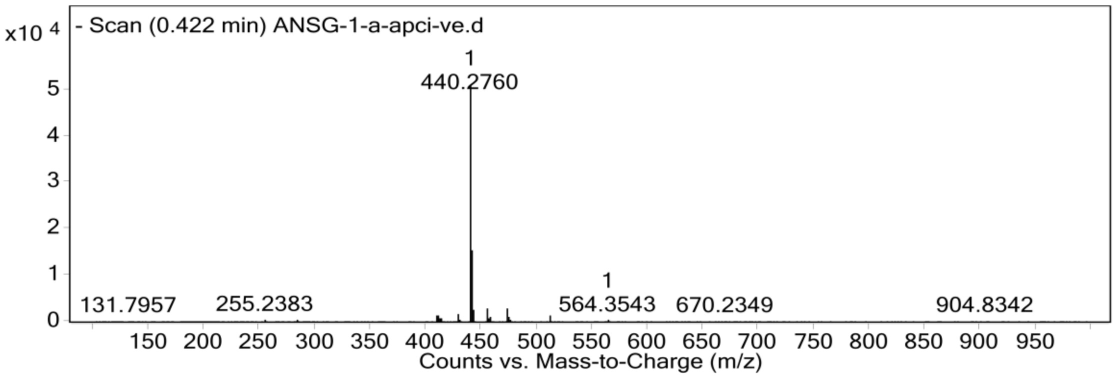

Figure S28. APCI-HR-MS spectrum of **PmDI**.

checkCIF/PLATON report

Structure factors have been supplied for datablock(s) shelx

THIS REPORT IS FOR GUIDANCE ONLY. IF USED AS PART OF A REVIEW PROCEDURE FOR PUBLICATION, IT SHOULD NOT REPLACE THE EXPERTISE OF AN EXPERIENCED CRYSTALLOGRAPHIC REFEREE.

No syntax errors found. CIF dictionary Interpreting this report

Datablock: shelx

Bond precision: C-C = 0.0344 A Wavelength=0.71073

Cell:	a=5.4537(5)	b=38.527(4)	c=15.1042(15)
	alpha=90	beta=93.702(4)	gamma=90
Temperature:	300 K		

	Calculated	Reported
Volume	3167.0(5)	3167.0(5)
Space group	P 21/m	P 21/m
Hall group	-P 2yb	-P 2yb
Moiety formula	C26 H36 N2 O4, 0.5(C10 H12 I2)	?
Sum formula	C31 H42 I N2 O4	C3.35 H4.54 I0.11 N0.22 O0.16
Mr	633.57	64.17
Dx,g cm-3	1.329	1.245
Z	4	37
Mu (mm-1)	1.046	1.036
F000	1308.0	1228.0
F000'	1306.59	
h,k,lmax	6,45,17	6,45,17
Nref	5692	5689
Tmin,Tmax	0.847,0.950	0.518,0.746
Tmin'	0.847	

Correction method= # Reported T Limits: Tmin=0.518 Tmax=0.746
AbsCorr = MULTI-SCAN

Data completeness= 0.999 Theta(max)= 24.999

R(reflections)= 0.1088(3830) wR2(reflections)= 0.2799(5689)

S = 1.141 Npar= 319

The following ALERTS were generated. Each ALERT has the format
test-name_ALERT_alert-type_alert-level.
Click on the hyperlinks for more details of the test.

Alert level A

```
SHFSU01_ALERT_2_A  The absolute value of parameter shift to su ratio > 0.20
           Absolute value of the parameter shift to su ratio given   2.659
           Additional refinement cycles may be required.
PLAT080_ALERT_2_A Maximum Shift/Error ...........................      2.66 Why ?
PLAT241_ALERT_2_A High   'MainMol' Ueq as Compared to Neighbors of       C18 Check
PLAT241_ALERT_2_A High   'MainMol' Ueq as Compared to Neighbors of       C32 Check
PLAT242_ALERT_2_A Low    'MainMol' Ueq as Compared to Neighbors of       C14 Check
PLAT242_ALERT_2_A Low    'MainMol' Ueq as Compared to Neighbors of       C31 Check
PLAT413_ALERT_2_A Short Inter XH3 .. XHn     H33B     ..H33B     .      1.67 Ang.
                                          -2-x,1-y,2-z  =      3_367 Check
```

Alert level B

```
PLAT043_ALERT_1_B Calculated and Reported Mol. Weight Differ by ..     39.99 Check
PLAT213_ALERT_2_B Atom C32              has ADP max/min Ratio .....       4.5 prolat
PLAT213_ALERT_2_B Atom C33              has ADP max/min Ratio .....       4.6 prolat
PLAT220_ALERT_2_B NonSolvent   Resd 1  C   Ueq(max)/Ueq(min) Range      10.0 Ratio
PLAT220_ALERT_2_B NonSolvent   Resd 2  C   Ueq(max)/Ueq(min) Range      10.0 Ratio
PLAT242_ALERT_2_B Low    'MainMol' Ueq as Compared to Neighbors of       C17 Check
PLAT242_ALERT_2_B Low    'MainMol' Ueq as Compared to Neighbors of       C27 Check
PLAT242_ALERT_2_B Low    'MainMol' Ueq as Compared to Neighbors of       C30 Check
PLAT342_ALERT_3_B Low Bond Precision on  C-C Bonds ..............   0.03443 Ang.
PLAT412_ALERT_2_B Short Intra XH3 .. XHn     H17A     ..H19A     .      1.79 Ang.
                                                x,y,z  =      1_555 Check
PLAT412_ALERT_2_B Short Intra XH3 .. XHn     H31B     ..H33B     .      1.76 Ang.
                                                x,y,z  =      1_555 Check
```

Alert level C

```
ABSMU01_ALERT_1_C  The ratio of given/expected absorption coefficient lies
              outside the range 0.99 <> 1.01
           Calculated value of mu =     1.053
           Value of mu given      =     1.036
RINTA01_ALERT_3_C  The value of Rint is greater than 0.12
           Rint given   0.136
PLAT020_ALERT_3_C The Value of Rint is Greater Than 0.12 .........     0.136 Report
PLAT041_ALERT_1_C Calc. and Reported SumFormula    Strings  Differ    Please Check
PLAT068_ALERT_1_C Reported F000 Differs from Calcd (or Missing)...    Please Check
PLAT082_ALERT_2_C High R1 Value ..................................      0.11 Report
PLAT084_ALERT_3_C High wR2 Value (i.e. > 0.25) ...................      0.28 Report
PLAT213_ALERT_2_C Atom C28              has ADP max/min Ratio .....       3.3 prolat
PLAT218_ALERT_3_C Constrained U(ij) Components(s) for C13       .          6 Check
PLAT218_ALERT_3_C Constrained U(ij) Components(s) for C16       .          6 Check
PLAT218_ALERT_3_C Constrained U(ij) Components(s) for C14       .          6 Check
PLAT218_ALERT_3_C Constrained U(ij) Components(s) for C30       .          6 Check
```

```
PLAT218_ALERT_3_C Constrained U(ij) Components(s) for C31        .          6 Check
PLAT218_ALERT_3_C Constrained U(ij) Components(s) for C17        .          6 Check
PLAT222_ALERT_3_C NonSolvent Resd 1  H   Uiso(max)/Uiso(min) Range       10.0 Ratio
PLAT222_ALERT_3_C NonSolvent Resd 2  H   Uiso(max)/Uiso(min) Range       10.0 Ratio
PLAT230_ALERT_2_C Hirshfeld Test Diff for    C28      --C29      .        6.6 s.u.
PLAT234_ALERT_4_C Large Hirshfeld Difference C8       --C10      .       0.17 Ang.
PLAT234_ALERT_4_C Large Hirshfeld Difference N2       --C26      .       0.20 Ang.
PLAT234_ALERT_4_C Large Hirshfeld Difference C20      --C24      .       0.16 Ang.
PLAT242_ALERT_2_C Low    'MainMol' Ueq as Compared to Neighbors of        C12 Check
PLAT242_ALERT_2_C Low    'MainMol' Ueq as Compared to Neighbors of        C13 Check
PLAT242_ALERT_2_C Low    'MainMol' Ueq as Compared to Neighbors of        C28 Check
PLAT250_ALERT_2_C Large U3/U1 Ratio for Average U(i,j) Tensor ....        3.0 Note
PLAT250_ALERT_2_C Large U3/U1 Ratio for Average U(i,j) Tensor ....        2.7 Note
PLAT260_ALERT_2_C Large Average Ueq of Residue Including        O1      0.242 Check
PLAT260_ALERT_2_C Large Average Ueq of Residue Including        O3      0.279 Check
PLAT361_ALERT_2_C Long   C(sp3)-C(sp3) Bond  C27      - C28      .       1.65 Ang.
PLAT411_ALERT_2_C Short Inter H...H Contact  H18B     ..H32A     .       2.06 Ang.
                                                   1+x,y,z  =      1_655 Check
PLAT412_ALERT_2_C Short Intra XH3 .. XHn     H3A      ..H5B      .       1.89 Ang.
                                                     x,y,z  =      1_555 Check
PLAT413_ALERT_2_C Short Inter XH3 .. XHn     H19C     ..H19C     .       2.03 Ang.
                                                -1-x,1-y,1-z  =      3_466 Check
PLAT906_ALERT_3_C Large K Value in the Analysis of Variance ......     20.196 Check
PLAT906_ALERT_3_C Large K Value in the Analysis of Variance ......      3.897 Check
PLAT906_ALERT_3_C Large K Value in the Analysis of Variance ......      2.103 Check
PLAT972_ALERT_2_C Check Calcd Resid. Dens.  1.12Ang From C20            -2.29 eA-3
```

Alert level G

```
FORMU01_ALERT_2_G  There is a discrepancy between the atom counts in the
            _chemical_formula_sum and the formula from the _atom_site* data.
            Atom count from _chemical_formula_sum:C3.35 H4.54 I0.11 N0.22 O0.16
            Atom count from the _atom_site data:  C3.351351 H4.540540 I0.108108 N0
CELLZ01_ALERT_1_G Difference between formula and atom_site contents detected.
CELLZ01_ALERT_1_G ALERT: Large difference may be due to a
            symmetry error - see SYMMG tests
           From the CIF: _cell_formula_units_Z   37
           From the CIF: _chemical_formula_sum  C3.35 H4.54 I0.11 N0.22 O0.16
           TEST: Compare cell contents of formula and atom_site data

           atom    Z*formula  cif sites diff
           C        123.95    124.00   -0.05
           H        167.98    168.00   -0.02
           I          4.07      4.00    0.07
           N          8.14      8.00    0.14
           O          5.92     16.00  -10.08
PLAT002_ALERT_2_G Number of Distance or Angle Restraints on AtSite         14 Note
PLAT003_ALERT_2_G Number of Uiso or Uij Restrained non-H Atoms ...         10 Report
PLAT045_ALERT_1_G Calculated and Reported Z Differ by a Factor ...      0.108 Check
PLAT083_ALERT_2_G SHELXL Second Parameter in WGHT  Unusually Large      31.85 Why ?
PLAT172_ALERT_4_G The CIF-Embedded .res File Contains DFIX Records         15 Report
PLAT177_ALERT_4_G The CIF-Embedded .res File Contains DELU Records          6 Report
PLAT178_ALERT_4_G The CIF-Embedded .res File Contains SIMU Records          6 Report
PLAT343_ALERT_2_G Unusual sp3      Angle Range in Main Residue for        C14 Check
PLAT431_ALERT_2_G Short Inter HL..A Contact  I001     ..O1       .       3.16 Ang.
                                                     x,y,z  =      1_555 Check
PLAT432_ALERT_2_G Short Inter X...Y Contact  O2       ..C21      .       2.93 Ang.
```

```
                                              x,y,z  =      1_555 Check
PLAT432_ALERT_2_G Short Inter X...Y Contact  O3       ..C6       .       2.91 Ang.
                                            -1+x,y,z  =      1_455 Check
PLAT720_ALERT_4_G Number of Unusual/Non-Standard Labels .........        1 Note
PLAT767_ALERT_4_G INS Embedded LIST 6 Instruction Should be LIST 4     Please Check
PLAT790_ALERT_4_G Centre of Gravity not Within Unit Cell: Resd.  #         2 Note
            C26 H36 N2 O4
PLAT793_ALERT_4_G Model has Chirality at C13         (Centro SPGR)         S Verify
PLAT793_ALERT_4_G Model has Chirality at C27         (Centro SPGR)         R Verify
PLAT860_ALERT_3_G Number of Least-Squares Restraints ............        51 Note
PLAT883_ALERT_1_G No Info/Value for _atom_sites_solution_primary .     Please Do !
PLAT910_ALERT_3_G Missing # of FCF Reflection(s) Below Theta(Min).        4 Note
PLAT965_ALERT_2_G The SHELXL WEIGHT Optimisation has not Converged     Please Check
PLAT967_ALERT_5_G Note: Two-Theta Cutoff Value in Embedded .res ..      50.0 Degree
PLAT978_ALERT_2_G Number C-C Bonds with Positive Residual Density.         0 Info
```

---

 7 **ALERT level A** = Most likely a serious problem - resolve or explain
11 **ALERT level B** = A potentially serious problem, consider carefully
35 **ALERT level C** = Check. Ensure it is not caused by an omission or oversight
25 **ALERT level G** = General information/check it is not something unexpected

 8 ALERT type 1 CIF construction/syntax error, inconsistent or missing data
41 ALERT type 2 Indicator that the structure model may be wrong or deficient
17 ALERT type 3 Indicator that the structure quality may be low
11 ALERT type 4 Improvement, methodology, query or suggestion
 1 ALERT type 5 Informative message, check

It is advisable to attempt to resolve as many as possible of the alerts in all categories. Often the minor alerts point to easily fixed oversights, errors and omissions in your CIF or refinement strategy, so attention to these fine details can be worthwhile. In order to resolve some of the more serious problems it may be necessary to carry out additional measurements or structure refinements. However, the purpose of your study may justify the reported deviations and the more serious of these should normally be commented upon in the discussion or experimental section of a paper or in the "special_details" fields of the CIF. checkCIF was carefully designed to identify outliers and unusual parameters, but every test has its limitations and alerts that are not important in a particular case may appear. Conversely, the absence of alerts does not guarantee there are no aspects of the results needing attention. It is up to the individual to critically assess their own results and, if necessary, seek expert advice.

Publication of your CIF in IUCr journals

A basic structural check has been run on your CIF. These basic checks will be run on all CIFs submitted for publication in IUCr journals (*Acta Crystallographica*, *Journal of Applied Crystallography*, *Journal of Synchrotron Radiation*); however, if you intend to submit to *Acta Crystallographica Section C* or *E* or *IUCrData*, you should make sure that full publication checks are run on the final version of your CIF prior to submission.

Publication of your CIF in other journals

Please refer to the *Notes for Authors* of the relevant journal for any special instructions relating to CIF submission.

PLATON version of 18/05/2022; check.def file version of 17/05/2022

Datablock shelx - ellipsoid plot

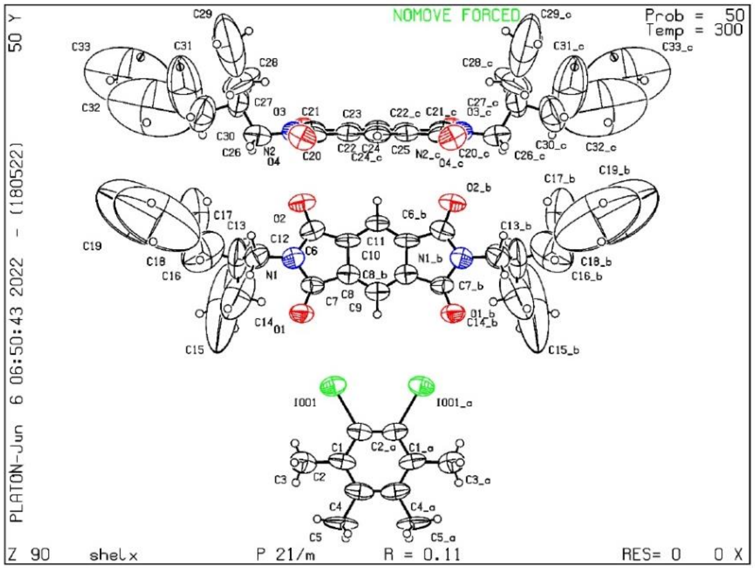

Figure S29. Check CIF documents for **A+D$_2$** co-crystal.

checkCIF/PLATON report

Structure factors have been supplied for datablock(s) shelx

THIS REPORT IS FOR GUIDANCE ONLY. IF USED AS PART OF A REVIEW PROCEDURE FOR PUBLICATION, IT SHOULD NOT REPLACE THE EXPERTISE OF AN EXPERIENCED CRYSTALLOGRAPHIC REFEREE.

No syntax errors found. CIF dictionary Interpreting this report

Datablock: shelx

Bond precision: C-C = 0.0300 A Wavelength=0.71073

Cell:	a=5.6900(4)	b=8.3410(6)	c=17.5111(12)
	alpha=79.981(3)	beta=82.483(2)	gamma=89.991(2)
Temperature:	299 K		

	Calculated	Reported
Volume	811.16(10)	811.16(10)
Space group	P 1	P 1
Hall group	P 1	P 1
Moiety formula	C26 H36 N2 O4, C6 H4 Br I	?
Sum formula	C32 H40 Br I N2 O4	C4 H5 Br0.12 I0.12 N0.25 O0.50
Mr	723.46	90.43
Dx,g cm-3	1.481	1.481
Z	1	8
Mu (mm-1)	2.255	2.255
F000	366.0	366.0
F000’	365.37	
h,k,lmax	6,9,20	6,9,20
Nref	5690[2845]	5650
Tmin,Tmax	0.805,0.854	0.518,0.746
Tmin’	0.595	

Correction method= # Reported T Limits: Tmin=0.518 Tmax=0.746
AbsCorr = MULTI-SCAN

Data completeness= 1.99/0.99 Theta(max)= 24.995

R(reflections)= 0.0510(4582) wR2(reflections)= 0.1570(5650)

S = 1.014 Npar= 344

The following ALERTS were generated. Each ALERT has the format
test-name_ALERT_alert-type_alert-level.
Click on the hyperlinks for more details of the test.

Alert level A

```
SHFSU01_ALERT_2_A  The absolute value of parameter shift to su ratio > 0.20
            Absolute value of the parameter shift to su ratio given   0.429
            Additional refinement cycles may be required.
PLAT080_ALERT_2_A Maximum Shift/Error ............................       0.43 Why ?
```

Alert level B

```
PLAT035_ALERT_1_B _chemical_absolute_configuration Info  Not Given     Please Do !
PLAT220_ALERT_2_B NonSolvent   Resd 1  C   Ueq(max)/Ueq(min) Range        6.7 Ratio
PLAT234_ALERT_4_B Large Hirshfeld Difference C24      --C25      .       0.28 Ang.
PLAT241_ALERT_2_B High   'MainMol' Ueq as Compared to Neighbors of         C5 Check
PLAT242_ALERT_2_B Low    'MainMol' Ueq as Compared to Neighbors of         C4 Check
PLAT242_ALERT_2_B Low    'MainMol' Ueq as Compared to Neighbors of        C25 Check
PLAT334_ALERT_2_B Small <C-C> Benzene Dist.   C27      -C32      .       1.34 Ang.
PLAT342_ALERT_3_B Low Bond Precision on  C-C Bonds ...............    0.03004 Ang.
PLAT410_ALERT_2_B Short Intra H...H Contact  H8B      ..H9B      .       1.87 Ang.
                                                      x,y,z  =      1_555 Check
PLAT926_ALERT_1_B Reported and Calculated   R1 Differ by .........     0.0059 Check
PLAT927_ALERT_1_B Reported and Calculated  wR2 Differ by .........     0.0138 Check
```

Alert level C

```
ABSMU01_ALERT_1_C  The ratio of given/expected absorption coefficient lies
               outside the range 0.99 <> 1.01
            Calculated value of mu =     2.168
            Value of mu given      =     2.255
CHEMW01_ALERT_1_C  The ratio of given/expected molecular weight as calculated
            from the _chemical_formula_sum lies outside
            the range 0.99 <> 1.01
            Calculated formula weight =    89.4019
            Formula weight given      =    90.4300
CHEMW01_ALERT_1_C  The difference between the given and expected weight for
            compound is greater 1 mass unit. Check that all hydrogen
            atoms have been taken into account.
STRVA01_ALERT_4_C           Flack test results are ambiguous.
           From the CIF: _refine_ls_abs_structure_Flack    0.502
           From the CIF: _refine_ls_abs_structure_Flack_su    0.004
PLAT041_ALERT_1_C Calc. and Reported SumFormula    Strings  Differ     Please Check
PLAT213_ALERT_2_C Atom C3              has ADP max/min Ratio .....        3.3 oblate
PLAT213_ALERT_2_C Atom C5              has ADP max/min Ratio .....        3.4 prolat
PLAT213_ALERT_2_C Atom C23             has ADP max/min Ratio .....        3.1 prolat
PLAT222_ALERT_3_C NonSolvent Resd 1  H   Uiso(max)/Uiso(min) Range        6.7 Ratio
PLAT234_ALERT_4_C Large Hirshfeld Difference O2       --C1       .       0.18 Ang.
PLAT234_ALERT_4_C Large Hirshfeld Difference N1       --C1       .       0.16 Ang.
PLAT234_ALERT_4_C Large Hirshfeld Difference N2       --C15      .       0.17 Ang.
PLAT234_ALERT_4_C Large Hirshfeld Difference C1       --C11      .       0.21 Ang.
PLAT234_ALERT_4_C Large Hirshfeld Difference C11      --C12      .       0.18 Ang.
PLAT234_ALERT_4_C Large Hirshfeld Difference C13      --C14      .       0.17 Ang.
PLAT234_ALERT_4_C Large Hirshfeld Difference C16      --C17      .       0.17 Ang.
```

```
PLAT234_ALERT_4_C Large Hirshfeld Difference C19      --C20      .       0.22 Ang.
PLAT234_ALERT_4_C Large Hirshfeld Difference I01      --C27      .       0.17 Ang.
PLAT234_ALERT_4_C Large Hirshfeld Difference Br01     --C30      .       0.19 Ang.
PLAT234_ALERT_4_C Large Hirshfeld Difference C28      --C29      .       0.20 Ang.
PLAT234_ALERT_4_C Large Hirshfeld Difference C31      --C32      .       0.24 Ang.
PLAT241_ALERT_2_C High   'MainMol' Ueq as Compared to Neighbors of         C3 Check
PLAT241_ALERT_2_C High   'MainMol' Ueq as Compared to Neighbors of         C9 Check
PLAT241_ALERT_2_C High   'MainMol' Ueq as Compared to Neighbors of        C23 Check
PLAT241_ALERT_2_C High   'MainMol' Ueq as Compared to Neighbors of        C24 Check
PLAT242_ALERT_2_C Low    'MainMol' Ueq as Compared to Neighbors of        C19 Check
PLAT244_ALERT_4_C Low    'Solvent' Ueq as Compared to Neighbors of        C27 Check
PLAT360_ALERT_2_C Short  C(sp3)-C(sp3) Bond  C8       - C9       .       1.39 Ang.
PLAT360_ALERT_2_C Short  C(sp3)-C(sp3) Bond  C9       - C10      .       1.42 Ang.
PLAT360_ALERT_2_C Short  C(sp3)-C(sp3) Bond  C21      - C22      .       1.42 Ang.
PLAT361_ALERT_2_C Long   C(sp3)-C(sp3) Bond  C5       - C6       .       1.71 Ang.
PLAT369_ALERT_2_C Long   C(sp2)-C(sp2) Bond  C14      - C15      .       1.56 Ang.
PLAT410_ALERT_2_C Short Intra H...H Contact  H23A     ..H24A     .       1.93 Ang.
                                                      x,y,z  =      1_555 Check
PLAT918_ALERT_3_C Reflection(s) with I(obs) much Smaller I(calc) .          2 Check
PLAT977_ALERT_2_C Check Negative Difference Density on H20       .      -0.31 eA-3
PLAT987_ALERT_1_C The Flack x is >> 0 -  Do a BASF/TWIN Refinement     Please Check
```

Alert level G

```
CELLZ01_ALERT_1_G Difference between formula and atom_site contents detected.
CELLZ01_ALERT_1_G ALERT: check formula stoichiometry or atom site occupancies.
           From the CIF: _cell_formula_units_Z    8
           From the CIF: _chemical_formula_sum  C4 H5 Br0.12 I0.12 N0.25 O0.50
           TEST: Compare cell contents of formula and atom_site data

           atom    Z*formula  cif sites diff
           C         32.00     32.00    0.00
           H         40.00     40.00    0.00
           Br         0.96      1.00   -0.04
           I          0.96      1.00   -0.04
           N          2.00      2.00    0.00
           O          4.00      4.00    0.00
PLAT002_ALERT_2_G Number of Distance or Angle Restraints on AtSite         14 Note
PLAT003_ALERT_2_G Number of Uiso or Uij Restrained non-H Atoms ...         12 Report
PLAT033_ALERT_4_G Flack x Value Deviates > 3.0 * sigma from Zero .      0.502 Note
PLAT045_ALERT_1_G Calculated and Reported Z Differ by a Factor ...      0.125 Check
PLAT111_ALERT_2_G ADDSYM Detects New (Pseudo) Centre of Symmetry .         95 %Fit
PLAT113_ALERT_2_G ADDSYM Suggests Possible Pseudo/New  Space Group        P-1 Check
PLAT172_ALERT_4_G The CIF-Embedded .res File Contains DFIX Records         10 Report
PLAT177_ALERT_4_G The CIF-Embedded .res File Contains DELU Records          8 Report
PLAT178_ALERT_4_G The CIF-Embedded .res File Contains SIMU Records          8 Report
PLAT335_ALERT_2_G Check Large C6 Ring C-C Range C11      -C18            0.24 Ang.
PLAT343_ALERT_2_G Unusual sp3      Angle Range in Main Residue for         C8 Check
PLAT343_ALERT_2_G Unusual sp3      Angle Range in Main Residue for         C9 Check
PLAT343_ALERT_2_G Unusual sp3      Angle Range in Main Residue for        C23 Check
PLAT343_ALERT_2_G Unusual sp3      Angle Range in Main Residue for        C24 Check
PLAT720_ALERT_4_G Number of Unusual/Non-Standard Labels ..........          5 Note
PLAT767_ALERT_4_G INS Embedded LIST 6 Instruction Should be LIST 4     Please Check
PLAT773_ALERT_2_G Check long C-C Bond in CIF: C5       --C6              1.71 Ang.
PLAT790_ALERT_4_G Centre of Gravity not Within Unit Cell: Resd.  #          2 Note
              C6 H4 Br I
PLAT791_ALERT_4_G Model has Chirality at C4          (Sohnke SpGr)          R Verify
```

```
PLAT791_ALERT_4_G Model has Chirality at C20         (Sohnke SpGr)          R Verify
PLAT860_ALERT_3_G Number of Least-Squares Restraints .............         60 Note
PLAT883_ALERT_1_G No Info/Value for _atom_sites_solution_primary .     Please Do !
PLAT909_ALERT_3_G Percentage of I>2sig(I) Data at Theta(Max) Still        51% Note
PLAT910_ALERT_3_G Missing # of FCF Reflection(s) Below Theta(Min).          1 Note
PLAT965_ALERT_2_G The SHELXL WEIGHT Optimisation has not Converged     Please Check
PLAT967_ALERT_5_G Note: Two-Theta Cutoff Value in Embedded .res ..      50.0 Degree
PLAT978_ALERT_2_G Number C-C Bonds with Positive Residual Density.          2 Info
```

```
  2 ALERT level A = Most likely a serious problem - resolve or explain
 11 ALERT level B = A potentially serious problem, consider carefully
 36 ALERT level C = Check. Ensure it is not caused by an omission or oversight
 29 ALERT level G = General information/check it is not something unexpected

 12 ALERT type 1 CIF construction/syntax error, inconsistent or missing data
 35 ALERT type 2 Indicator that the structure model may be wrong or deficient
  6 ALERT type 3 Indicator that the structure quality may be low
 24 ALERT type 4 Improvement, methodology, query or suggestion
  1 ALERT type 5 Informative message, check
```

It is advisable to attempt to resolve as many as possible of the alerts in all categories. Often the minor alerts point to easily fixed oversights, errors and omissions in your CIF or refinement strategy, so attention to these fine details can be worthwhile. In order to resolve some of the more serious problems it may be necessary to carry out additional measurements or structure refinements. However, the purpose of your study may justify the reported deviations and the more serious of these should normally be commented upon in the discussion or experimental section of a paper or in the "special_details" fields of the CIF. checkCIF was carefully designed to identify outliers and unusual parameters, but every test has its limitations and alerts that are not important in a particular case may appear. Conversely, the absence of alerts does not guarantee there are no aspects of the results needing attention. It is up to the individual to critically assess their own results and, if necessary, seek expert advice.

Publication of your CIF in IUCr journals

A basic structural check has been run on your CIF. These basic checks will be run on all CIFs submitted for publication in IUCr journals (*Acta Crystallographica*, *Journal of Applied Crystallography*, *Journal of Synchrotron Radiation*); however, if you intend to submit to *Acta Crystallographica Section C* or *E* or *IUCrData*, you should make sure that full publication checks are run on the final version of your CIF prior to submission.

Publication of your CIF in other journals

Please refer to the *Notes for Authors* of the relevant journal for any special instructions relating to CIF submission.

PLATON version of 18/05/2022; check.def file version of 17/05/2022

Datablock shelx - ellipsoid plot

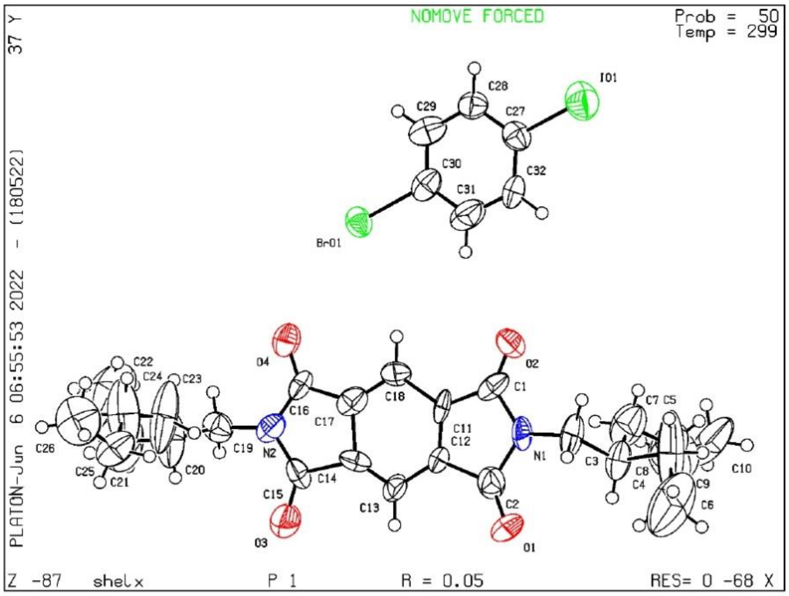

Figure S30. Check CIF documents for **A+D$_4$** co-crystal.

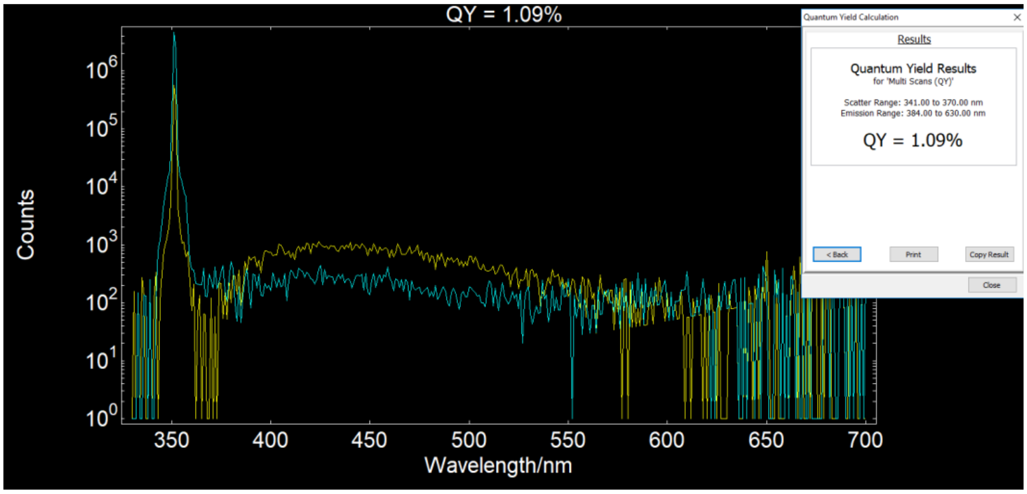

Figure S31. Screenshots of phosphorescence quantum yield for **A** crystal.

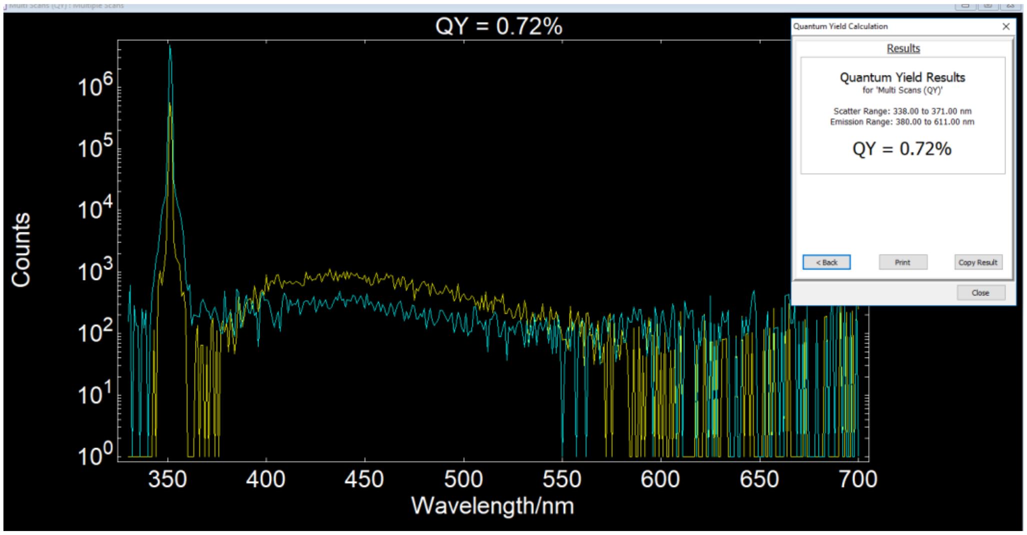

Figure S32. Screenshots of phosphorescence quantum yield for **D**$_1$ crystal.

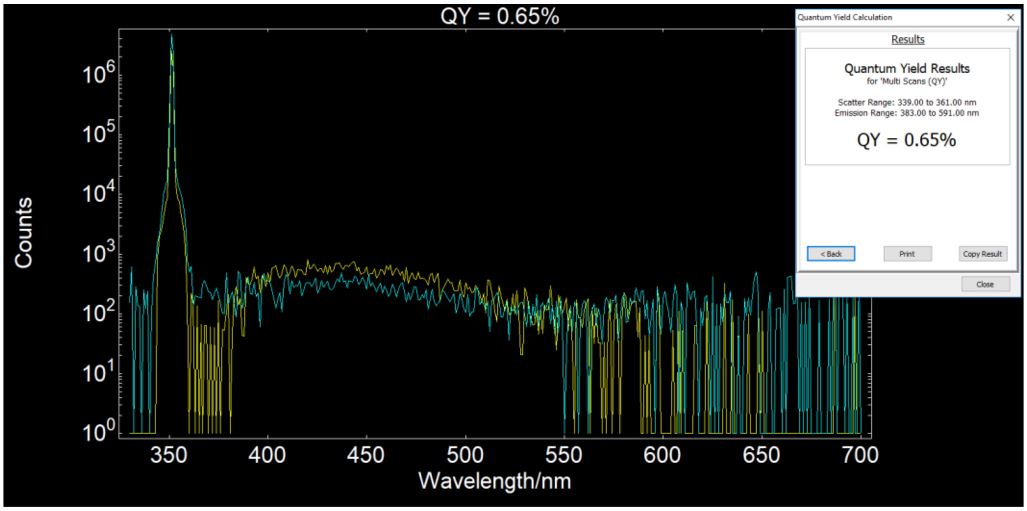

Figure S33. Screenshots of phosphorescence quantum yield for **D**$_2$ crystal.

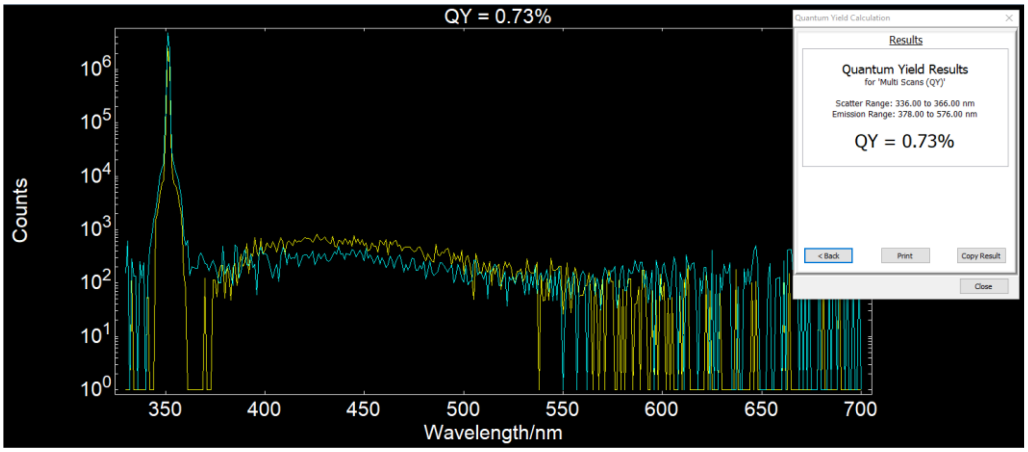

Figure S34. Screenshots of phosphorescence quantum yield for $\mathbf{D_3}$ crystal.

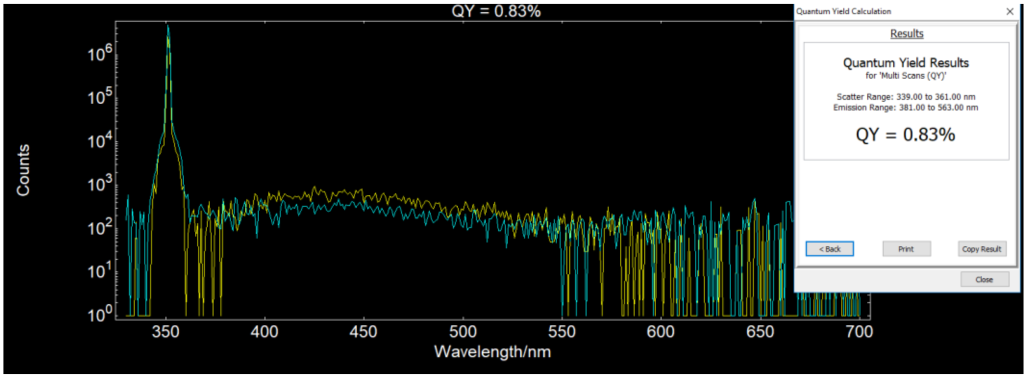

Figure S35. Screenshots of phosphorescence quantum yield for $\mathbf{D_4}$ crystal.

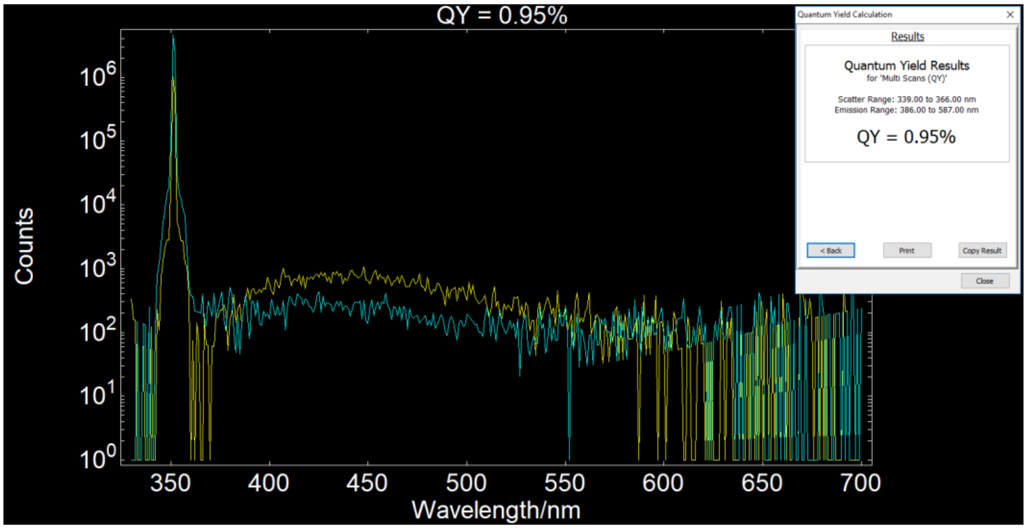

Figure S36. Screenshots of phosphorescence quantum yield for $\mathbf{D_5}$ crystal.

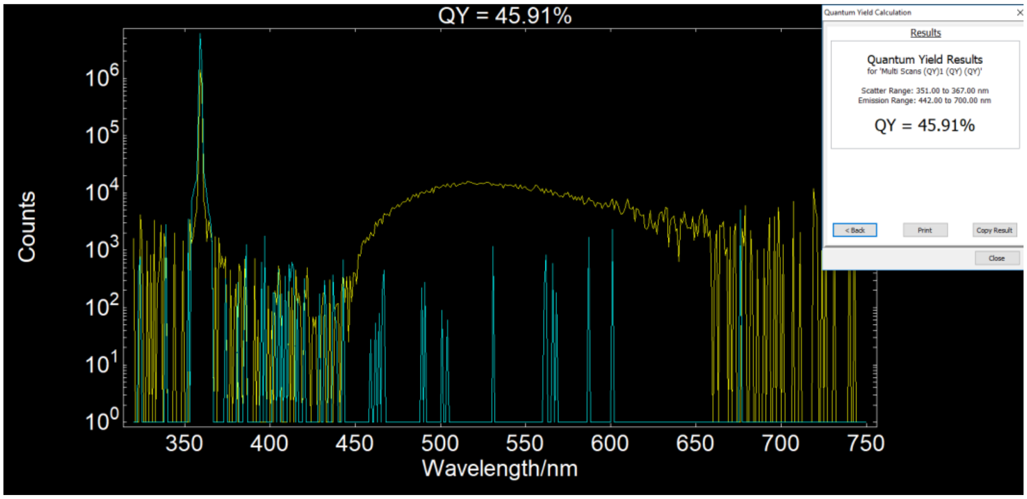

Figure S37. Screenshots of phosphorescence quantum yield for **A+D$_1$** co-crystal.

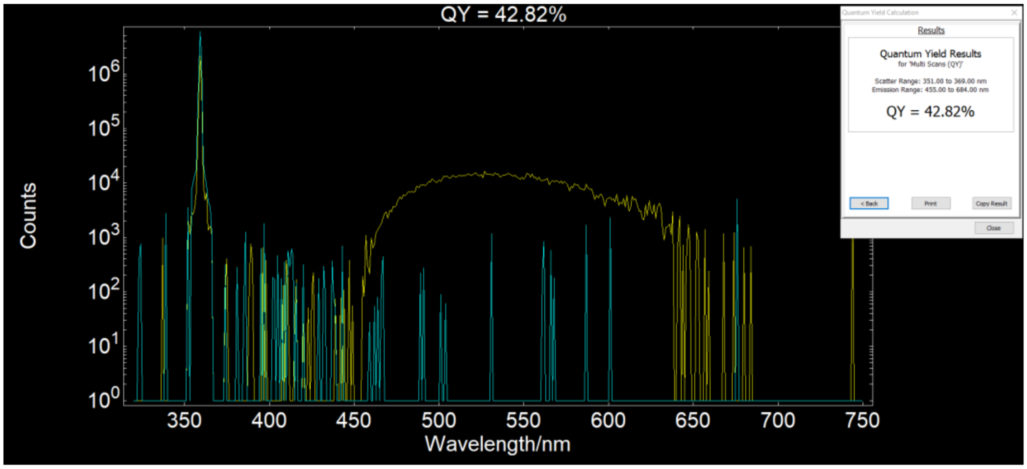

Figure S38. Screenshots of phosphorescence quantum yield for **A+D$_2$** co-crystal.

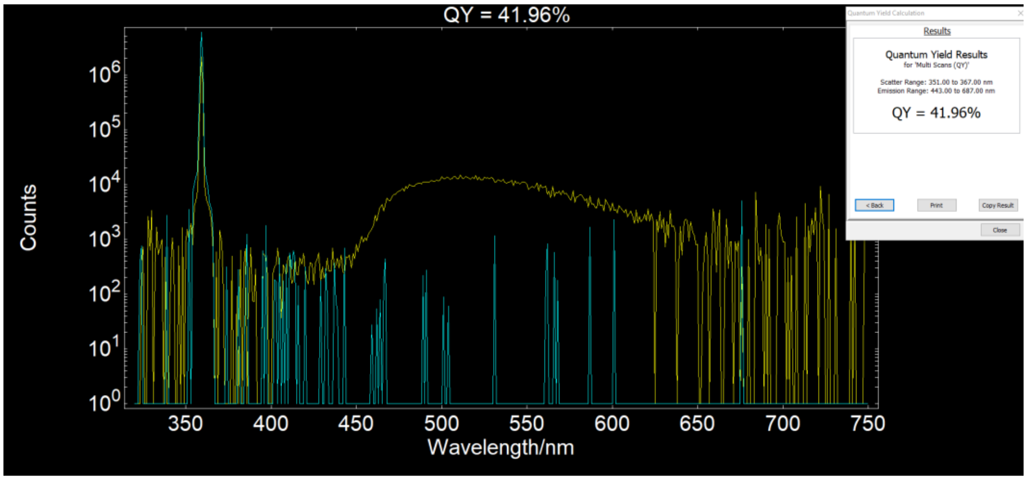

Figure S39. Screenshots of phosphorescence quantum yield for **A+D$_3$** co-crystal.

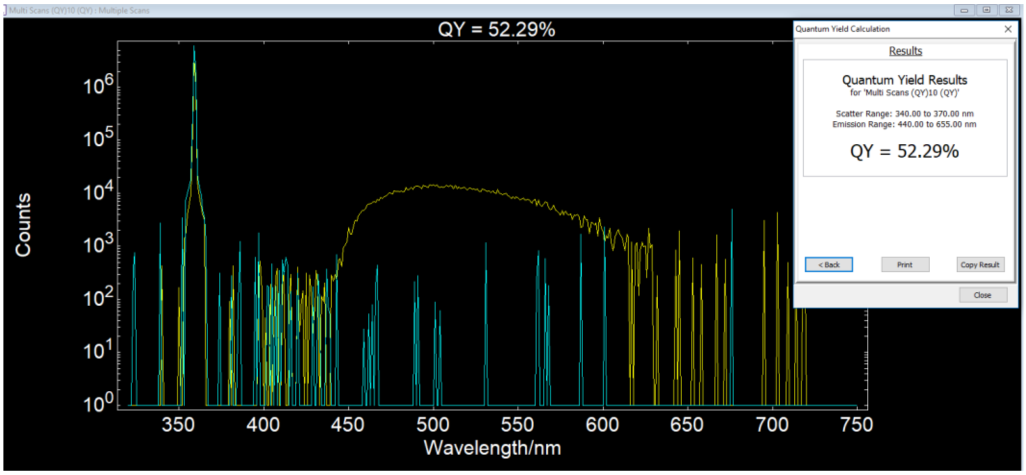

Figure S40. Screenshots of phosphorescence quantum yield for **A+D$_4$** co-crystal.

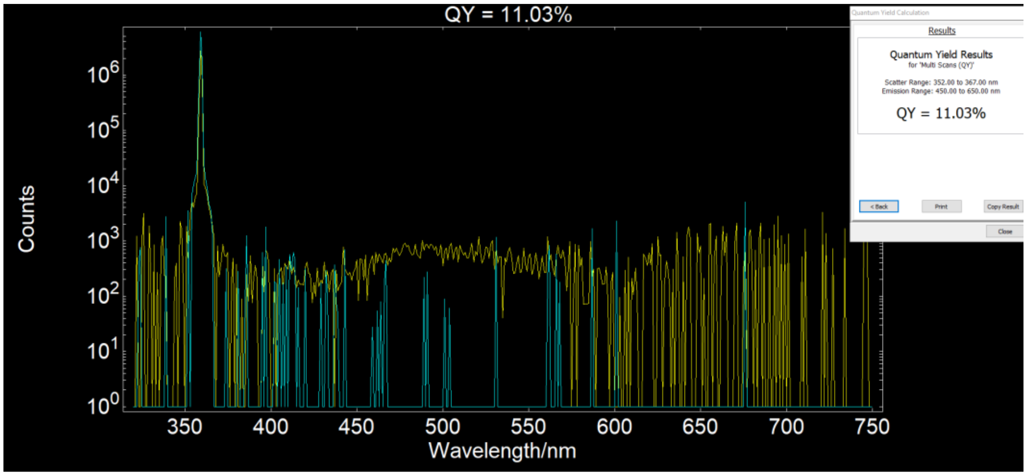

Figure S41. Screenshots of phosphorescence quantum yield for **A+D$_5$** co-crystal.

6. References

[S1] Gaussian 16, Revision C.01, M. J. Frisch, G. W. Trucks, H. B. Schlegel, G. E. Scuseria, M. A. Robb, J. R. Cheeseman, G. Scalmani, V. Barone, G. A. Petersson, H. Nakatsuji, X. Li, M. Caricato, A. V. Marenich, J. Bloino, B. G. Janesko, R. Gomperts, B. Mennucci, H. P. Hratchian, J. V. Ortiz, A. F. Izmaylov, J. L. Sonnenberg, D. Williams-Young, F. Ding, G. Lipparini, F. Egidi, J. Goings, B. Peng, A. Petrone, T. Henderson, D. Ranasinghe, V. G. Zakrzewski, J. Gao, N. Rega, G. Zheng, W. Liang, M. Hada, M. Ehara, K. Toyota, R. Fukuda, J. Hasegawa, M. Ishida, T. Nakajima, Y. Honda, O. Kitao, H. Nakai, T. Vreven, K. Throssell, J. A. Jr. Montgomery, J. E. Peralta, F. Ogliaro, M. J. Bearpark, J. J. Heyd, E. N. Brothers, K. N. Kudin, V. N. Staroverov, T. A. Keith, R. Kobayashi, J. Normand, K. Raghavachari, A. P. Rendell, J. C. Burant, S. S. Iyengar, J. Tomasi, M. Cossi, J. M. Millam, M. Klene, C, Adamo, R. Cammi, J. W. Ochterski, R. L. Martin, K. Morokuma, O. Farkas, J. B. Foresman and D. J. Fox, Gaussian, Inc., Wallingford CT, 2016.

[S2] A. D. Becke, *J. Chem. Phys.*1993, **98**, 1372-1377.

[S3] C. Lee,W. Yang and R. G. Parr. *Phys. Rev. B* 1988, **37**, 785-789.

[S4] B. Miehlich, A. Savin, H. Stoll and H. Preuss, *Chem.Phys. Lett.*1989, **157**, 200-206.

[S5] S. Hirata and M. Head-Gordon, *Chem. Phys. Lett.* 1999, **314**, 291-299.

[S6] ADF2017, SCM, Theoretical Chemistry, VrijeUniversiteit, Amsterdam, The Netherlands, http://www.scm.com.

[S7] D. Cao, M. Hong, A. K. Blackburn, Z. Liu, J. M. Holcroft and J. F. Stoddart, J. F. *Chem. Sci.* 2014, **5**, 4242-4248.

[S8] S. M. Luo, K. A. Stellmach, S. M. Ikuzwe and D. D. Cao, *J. Org. Chem.* 2019, **84**, 10362-10370.

[S9] X. Huang, M. Hu, X. Zhao, C. Li, Z. Yuan, X. Liu, C. Cai, Y. Zhang, Y. Hu and Y. Chen, *Org. Lett.* 2019, **21**, 3382-3386.
